# The Latitudinal Biotic Interaction Hypothesis revisited: contrasting latitudinal richness gradients in actively vs. passively accumulated interaction partners of honey bees

**DOI:** 10.1186/s12862-025-02363-1

**Published:** 2025-03-17

**Authors:** Alyssa R. Cirtwill, Tomas Roslin, Pablo Peña-Aguilera, Agathe Agboto, William Bercê, Svetlana N. Bondarchuk, Robert Brodschneider, Behzad Heidari, Camara Kaizirege, Justine Muhoro Nyaga, Ojonugwa Ekpah, Gonzalo Ossa Gomez, Claudia Paz, Christian Pirk, Amir Salehi-Najafabadi, Anneli Salonen, Chantal Soloniaina, Helena Wirta

**Affiliations:** 1 Carex EcoLogics, Bracebridge, ON Canada; 2 Department of Ecology, Swedish University of Agricultural Sciences, Uppsala, Sweden; 3 Organismal and Evolutionary Biology Research Programme, Faculty of Biological and Environmental Sciences, University of Helsinki, Helsinki, Finland; 4 University of Abomey-Calavi, Faculty of Agronomic Sciences, Laboratory of Agricultural Entomology, Abomey-Calavi, Benin; 5 Duilio Meliponicultura, Jundiaí, São Paulo Brazil; 6 Sikhote-Alin State Nature Biosphere Reserve Named After K.G. Abramov, 44 Partizanskaya Str., Terney, Primorsky Krai 692150 Russia; 7 Department of Biology, University of Graz, Graz, Austria; 8 IKBM, Department of Chemistry, Bioscience and Environmental Engineering, University of Stavanger, 4036 Stavanger, Norway; 9 Tanzifarm Tanzania Limited, Mlele District, Katavi Region Tanzania; 10 Department of Biological Sciences, University of Embu, Embu, Kenya; 11 Institute of Geoecology, Department Landscape Ecology and Environmental Systems Analysis, Technische Universität Braunschweig, Braunschweig, Germany; 12 ConserBat EIRL, San Fabian, Ñuble Chile; 13 Department of Biodiversity, Institute of Biosciences, São Paulo State University, Av 24A 1515, Rio Claro, SP 13506-900 Brazil; 14 Current address: Department of Entomology and Acarology, Laboratory of Pathology and Microbial Control, University of São Paulo, 13418-900 Piracicaba, SP Brazil; 15 Social Insects Research Group, Department of Zoology & Entomology, University of Pretoria, Pretoria, South Africa; 16 Department of Microbiology, School of Biology, College of Science, University of Tehran, Tehran, Iran; 17 Finnish Beekeepers’ Association, Ullanlinnankatu 1 A 3, 00130 Helsinki, Finland; 18 Department of International Relations and Partnership, Ministry of Education, Antananarivo, Madagascar; 19 Department of Ecology and Environmental Science, Umeå University, Umeå, Sweden; 20 Department of Agricultural Sciences, Faculty of Agriculture and Forestry, University of Helsinki, Helsinki, Finland

**Keywords:** Neutral theory, Bacteria, Pollination, Flowering plant, *Apis mellifera*, DNA metabarcoding

## Abstract

**Background:**

Contrasting hypotheses suggest that the number of biotic interactions per species could either increase towards the equator due to the increasing richness of potential interaction partners (Neutral theory), or decrease in the tropics due to increased biotic competition (Latitudinal Biotic Interaction Hypothesis). Empirical testing of these hypotheses remains limited due to practical limitations, differences in methodology, and species turnover across latitudes. Here, we focus on a single species with a worldwide distribution, the honey bee (*Apis mellifera* L.), to assess how the number of different types of interactions vary across latitudes. Foraging honey bees interact with many organisms in their local environment, including plants they actively select to visit and microbes that they largely encounter passively (i.e., unintentionally and more or less randomly). Tissue pieces and spores of these organisms are carried to the hive by foraging honey bees and end up preserved within honey, providing a rich record of the species honey bees encounter in nature.

**Results:**

Using honey samples from around the globe, we show that while honey bees visit more plant taxa at higher latitudes, they encounter more bacteria in the tropics.

**Conclusions:**

These different components of honey bees’ biotic niche support the latitudinal biotic interaction hypothesis for actively-chosen interactions, but are more consistent with neutral theory (assuming greater bacterial richness in the tropics) for unintentional interactions.

**Supplementary Information:**

The online version contains supplementary material available at 10.1186/s12862-025-02363-1.

## Background

Each species interacts with a subset of the other species present in its environment, but the size of that subset is expected to vary strongly with latitude [1]. In part, this is due to the general trend of greater species richness in the tropics for many taxa [2–7]. As a null model, Neutral Theory proposes that a species’ interaction partners are a random sample of those available [8]. Therefore species should have more interaction partners in the species-rich tropics. Alternatively, the Latitudinal Biotic Interaction Hypothesis posits that higher species richness and narrower ranges of abiotic conditions in the tropics may result in fewer, but stronger, biotic interactions towards the Equator [9–11]. Although there is some support for stronger herbivory, carnivory, and plant-pollinator mutualism towards the tropics [10, 12–15], other studies show little or no evidence of such a trend [16–20]. The generality of the relationship between biotic interactions and latitude thus remains debated.

An important reason for differences in findings are differences in methodology – not least in the differences in how biotic interactions are measured [14, 21]. The “strength” of biotic interactions has been described using metrics ranging from plant investment in secondary compounds through bite marks in play dough to counts of observed interactions [20, 22]). Moreover, studies comparing generalism in communities sampled over a large geographic area will unavoidably confound effects of changes in richness with effects of changes to the composition of the local species pool, especially where the true richness and/or composition of the local community is not known at all locations. Differences in species traits due to changes in community composition (such as a larger proportion of vertebrate pollinators in the tropics) could explain some cases of greater generalism in the tropics without referring to either Neutral Theory or the Latitudinal Biotic Interaction Hypothesis [20].

**Fig. 1:**
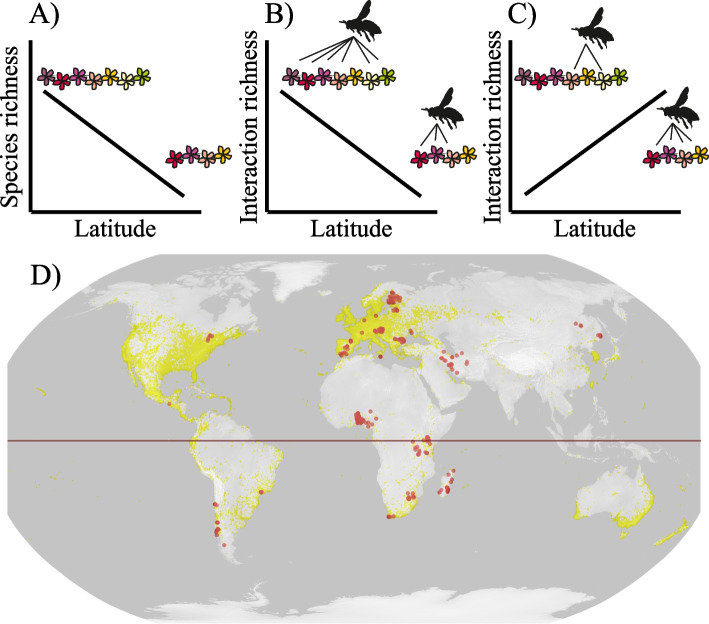
In general, flowering plant richness decreases with increasing latitude (**A**). However, the number of plants a focal species (here, the honey bee) interacts with (its niche breadth) could covary with this decline in multiple ways. If the species is a wide generalist, with interactions representing a random sample of the potential partners, then the niche breadth should follow the pattern of species richness aligning with the Neutral Theory (**B**). Alternatively, the species may focus on fewer species in the tropics but interact more strongly with each species, as proposed by the Latitudinal Biotic Interaction Hypothesis. This would lead to greater generalism at higher latitudes (**C**). We use the honey bee *Apis mellifera* as our focus to explore latitudinal gradients in niche breadth because of the global distribution of this species - as seen in global distribution data available through GBIF (**D**; [26]) shown by the yellow dots on the map. Note that *A. mellifera*’s occurrence is more comprehensive than shown by GBIF records, covering most of the world [27]. In particular, note that *A. mellifera* occurs, and our dataset includes records from, much more of Africa than is included in the GBIF record. The red dots indicate the location of samples used in the current study (**D**)

One way to resolve some of these methodological issues is to characterize the set of interaction partners of a single focal species with a near-global distribution, yielding a truly comparable measure of niche breadth over latitude. The honey bee (*Apis mellifera* L.) offers an ideal target for this type of a study. Native to Africa, most of Europe, and the Middle East [23–25], this species has been anthropogenically introduced to widely variable biotic settings around the world ([26]; Fig. 1D). *Apis mellifera* shows several evolutionary lineages, each with multiple subspecies. One of these lineages covers most of Africa, while others occur in Europe and in the Middle East [25, 27]. Despite the many subspecies with adaptations to different environments, most managed honey bees are mixtures of different subspecies [28]. Moreover, despite differences among subspecies, all *A. mellifera* show the same colony-level behaviour with thousands of foragers collecting nectar and pollen for feeding the adults, rearing larvae, and storage for times of resource shortage [23]. This stored honey provides a representative sample of honey bees’ interactions with other species.

Honey harvesting by beekeepers typically takes place after an active foraging season of the honey bees, before a period of dearth due to cold, drought, or excessive rains. On the other hand, honey bees consume honey during inactive periods. Honey is also consumed during the active foraging season, whenever nectar availability is lower or resource demand is high. Thus, stored honey provides a time-integrated sample of the colony’s interactions over a time period extending over approximately the past two months [23, 29, 30]. As well as providing convenient interaction sampling, the honey bee is an important model system because of its immense importance as a crop and wild plant pollinator [31, 32].

Importantly, not all interactions are alike: some are actively sought out by at least one of the species involved, whereas others represent chance encounters with other taxa. In this context, honey bees allow us to test whether the Latitudinal Biotic Interaction Hypothesis holds for different types of interactions. Honey bees actively select a variety of plants to forage on, for both nectar and pollen, from the available flowering plant pool [29, 33–35].

Bee interactions with microbes are more varied: honey bees interact with microbes in their guts [36, 37], in hives [38, 39], on flowers [40, 41], and in the broader environment, including air, water, and soil. Honey bees encounter these microbes while walking on soil, drinking from various water sources, scouting and foraging [42, 43], or more rarely inherit them within the hive [36, 37]. These microbes have a variety of effects on honey bees, but are generally encountered without being selected by the honey bees [44–46]. The obvious exception are five species of gut bacteria that are passed down within the colony [36, 37]. Conveniently, both honey bee-plant and honey bee-microbe interactions can be resolved by examining DNA traces in honey samples [30, 46–48]. This offers a common methodology for measuring bee interactions with both plants and bacteria.

The distinction between different ways of accumulating interaction partners (above) allows us to generate a series of predictions. Overall, we expect to find different trends in the number of interaction partners with respect to each group of interaction partners, since honey bees tend to choose plant partners actively while encountering most bacteria passively. For flowering plants, species richness generally increases towards the tropics [49] together with increasing primary productivity [5, 50]. Therefore, the number of plant species found in honey can be used to test the scenarios described in Fig. 1. If honey bees interact with more plant species as richness increases (Fig. 1A), that would translate into increasing generalism towards the tropics (Fig. 1B), and honey from the tropics should include DNA from more plants. In contrast, if specialization increases due to higher competition at lower latitudes, then we would expect honey bees to interact with fewer plant species in the tropics (specialisation, Fig. 1C).

Among microbes, latitudinal trends in richness are poorly explored, with no global synthesis available to date (but see [51–56]). Early reports show varying trends for different taxonomic or functional groups of microbes [57–59], but overall, we may assume greater bacterial richness at lower latitudes following the trend in other organism groups due to higher productivity towards the equator [5, 50]. As most interactions between honey bees and bacteria will result from random encounters in their environment (“environmental sampling”; [42, 43]), the system should conform with Neutral Theory (Fig. 1B; [8]). That is, we expect the number of bacteria present in honey to be a consistent proportion, though not a complete record, of true bacterial richness [46, 48]. If the richness of microbes increases with decreasing latitude, then we may predict a higher overall number of microbial interaction partners towards the tropics. The obvious exception should be gut bacteria, which are actively passed on through the colony [36, 37].

In this study we use a global collection of DNA extracted from honey samples to test the hypotheses advanced above, and thereby answer the following questions: 1) Does the niche breadth of the honey bee vary over latitude in terms of the plants selected to visit? 2) Does the niche breadth of the honey bee vary over latitude in terms of the bacteria inherited and randomly encountered? 3) Do latitudinal trends vary among bacterial groups, given earlier reports of differential trends in different bacterial clades? To search for such variation, we compare bacterial families containing genera and species strongly associated with honey bees and that were well-represented within our dataset.

## Methods

### Sampling, DNA laboratory and bioinformatic analyses

#### Samples

To study the plant usage and microbes encountered by the honeybees across different latitudes, we sourced honey samples from beekeepers from twenty-one countries across the world (Fig. 1; number of samples per country in Table *S1*). The samples presented honey harvested by one or multiple beekeepers from a region, within two to three months of active honey collecting by honeybees in each region. The number of hives from which the honey was harvested, along with the type of bee hives and honey extraction method, was recorded.

Before extracting the DNA, the samples were preprocessed. Two times 10 g of honey was diluted to 30 ml of DNA clean water (MilliQ, Merck KGaA, Germany) in a 50 ml tube. The honey was let to dissolve into the water for 30 min in 60°. The samples were then centrifuged at 8000 G for 60 min (Centrifuge 5810 R, Eppendorf, Germany), after which most of the supernatant was discarded and the pellet was transferred to a 2 ml tube. The 2 ml tube was further centrifuged at 11 000 G for 5 min and the remaining supernatant was removed. The preprocessed samples were stored in freezer until DNA extraction.

#### DNA extraction, target amplification, sequencing library preparation and sequencing

The total DNA was extracted with the DNeasy Plant Mini Kit (Qiagen, Germany), with following modifications. First, the pellet was resuspended in 400$\upmu$l of buffer AP1, and then 4$\upmu$l RNase, 4$\upmu$l proteinase K (20mg/ml, Macherey-Nagel) and one 3 mm tungsten carbide bead was added to each sample tube. The sample was then disrupted 2 x 2 min 30 Hz (Mixer Mill MM 400, Retsch, Germany). DNA extraction then followed the protocol with the exception of skipping the QIAshredder column step to avoid loss of DNA. With each batch of samples extracted, a blank DNA control was included. In the laboratory all the steps before the amplifications were done in a laminar hood wiped with ethanol and cleaned of DNA with 1 hour UV light every night. We only used DNA-free tubes, pipette tips, and PCR plates as well as DNA-free water.

The initial amplifications were done with a total volume of 10 $\upmu$l, each containing 5 $\upmu$l MyTaq Red Mix (Bioline, London, UK), 1.3 $\upmu$l DNA-free water, 0.3 $\upmu$l of each primer (10 $\upmu$M) and 3 $\upmu$l of DNA extract. PCR cycling conditions were as follows, with primer-specific annealing temperatures using tagged primers (Table *S2*), allowing the attachment of the sequencing primers and indexes in the second PCR. For plants with primers ITS2-F and ITS2-R [60, 61] annealing was at 47°C) and for bacteria with primers 16S_515FB and 16S_806RB [62, 63] annealing was at 50°C.

The initial denaturation was for 3 min at 95°C, followed by 28 cycles of 30 s at 95°C (denaturation), 30 s at 47–55°C (annealing), 30 s 72°C (extension), and ending with final extension for 7 min at 72°C. To minimize initial bias of amplification, each reaction was carried out as two replicates. All the amplicons were checked on a 1% agarose gel and imaged with a BioRad imager to check the reaction had worked and the DNA and PCR controls were clean. The PCR replicates were combined before library-PCR as 1.3 $\upmu$l of each PCR product replicate. Illumina-specific adapters and combinatorial indexing, unique dual-index combinations for each sample, was used [64]. The library PCR had a total volume of 10 $\upmu$l, each containing 5 $\upmu$l MyTaq Red Mix (Bioline, London, UK), 0.3 $\upmu$l of reverse primer (10 $\upmu$M), 2.1 $\upmu$l of forward primer (1.43 $\upmu$M) and 2.6 $\upmu$l of the locus-specific combined 1st PCR product. PCR cycling conditions were as follows, the same for all gene regions for the library PCR. Starting with 4 min at 95°C to denature, followed by 15 cycles of 20 s at 98°C, 15 s at 60°C and 30 s at 72°C, and ending with 3 min at 72°C. DNA libraries were pooled per gene region and per 96 samples, and concentrated using a SPRI bead protocol. The concentrated pooled sample was loaded on 1% agarose gel (Agarose tablets + TAE) and run with 90 V for 120 minutes. The target bands were cut on UV light and the pooled sample was cleaned from gel with the PCR and Gel CleanUp Kit (Macherey-Nagel), diluted in 2 x 20 $\upmu$l of the elution buffer provided in the kit. The DNA concentration of the cleaned pools were measured with Qubit 2.0 (dsHS DNA Kit, ThermoFisher Scientific).

Based on the compatible lengths of the targeted gene regions, the pools of 96 samples were combined in equimolar ratios and sequenced in three MiSeq sequencing runs with v3 chemistry with 600 cycles and 2 x 300 bp paired-end read length.

#### Bioinformatics

Bioinformatics processing of reads followed Kaunisto et al. [65]. For the bioinformatics processing the reads of all samples were combined per gene region. The processing of reads was started by truncating the reads to 220 bp for 16S and 240 bp for ITS2. This was done to cut off lower quality ends before merging the paired ends for each gene region using VSEARCH [66] with a maximum of 80 differences allowed for overlap and a minimum assembly length of 150 bp. The merged reads were quality controlled by fastq_maxee, with maxee = 3. The merged and quality controlled reads were only retained if they contained the expected primers at each end. Primers were removed using cutadapt with a maximum of 0. 2 error rate for primers, and reads were kept with minimum length of 100 bp after primer removal. The reads were dereplicated and singletons were removed. The reads were denoised to zero-radius operational taxonomic units (ZOTU) using with unoise3 with USEARCH [66]. A ZOTU table was built and the taxonomic assignation of ZOTUs was done by comparison against a specific reference database for each gene region with VSEARCH. 16S for bacteria were compared against the 16S RDP reference database, version 18 [67], and ITS2 for plants against an ITS2 reference database from PLANTiTS, accessed 21.3.2022 [68]. The assignment of a ZOTU to a taxon was accepted if the SINTAX probability [69] was $\ge$ 0.9, at each taxonomic level.

To remove possible misassigned reads and false positives, due to contamination, we further filtered the reads in ZOTUs (following e.g., [70, 71]). As small numbers of reads were found in all controls, reads were removed if they were less than the maximum number of reads from the DNA extraction or PCR negative controls from all the samples for each ZOTU. ZOTUs with less than 0.05% of the total read number of that sample were removed, as well as ZOTUs with less than 10 reads were removed.

### Statistical methods overview

We were primarily interested in how honey bee niche breadths, as measured using plants (which are selected as resources to visit) and bacteria (mainly encountered haphazardly within the environment) vary over latitude. After determining that the other sample characteristics we collected covaried significantly with latitude (see Appendix *S2*) and that niche breadth covaried with the total number of DNA reads obtained from a sample (see Appendix *S3*), we chose to test the latitude-niche breadth relationship using quasi-Poisson regressions relating niche breadth (number of ZOTU, genera, or families identified in a sample) to absolute latitude and the log of total reads in the sample. We fit these regressions separately for plant and bacteria ZOTUs, genera, and families (six models) using the R [72] base function ‘glm’. To test whether these relationships differed between hemispheres, we fit an additional set of six models including absolute latitude, hemisphere, their interaction, and log of total reads. For plants (and bacteria), there were 167 (170) samples from the northern hemisphere and 84 (83) from the southern hemisphere.

Finally, because the richness trends of bacteria over latitude are not well-known and may differ between groups, we examined latitudinal gradients within a few focal families. We chose families that contain either bacterial genera or species with known strong associations with honey bees, such as beneficial or disease-causing taxa and that were well-represented in our dataset (i.e., were found in >50% of all samples). The families chosen were Acetobacteraceae, Clostridiaceae, Enterobacteriaceae, Enterococcaceae, Lactobacillaceae, Moraxellaceae, Orbaceae and Paenibacillaceae. *Parasaccharibacter abium* is a bacterium living commonly in hives and also other Acetobacteraceae species are associated with honey bees, being beneficial to them in fight against disease causing agents [73, 74]. *Clostridium botulinum*, a member of Clostridiaceae, is an environmental, pathogenic bacterium transported by honey bees and found commonly in honey [75]. Multiple species of the genus *Enterobacter* (Enterobacteriaceae) occur in honey bee guts and may be both beneficial or detrimental to honey bees, depending on the conditions [76]. Species of *Enterococcus* (Enterococcaceae) are often found in honey bee guts as well, likely contributing to digestion [77]. Two species of the genus *Lactobacillus* (Lactobacillaceae) are found ubiquitously in honey bee guts and one, *Apilactobacillus kunkeii*, commonly in honey as well as living in the nectar and nectar sacs [39, 46]. Within the family Moraxellaceae, the species *Acinetobacter apium* is found in bee guts [78] while other species of *Acinetobacter* are found in nectar [79]. *Frischella perrara* and *Gilliamella apicola* (Orbaceae) are also among the core members of the honey bee gut microbiota [39]. *Paenibacillus larvae*, representing the family Paenibacillaceae, is the bacterium causing a severe bee disease, the American foulbrood [80]. We thus fit an additional round of eight quasi-Poisson regressions relating the number of ZOTUs in each focal bacteria family to absolute latitude and the log of total reads.

## Results

### Data overview

Plant DNA was amplified and successfully sequenced from 251 honey samples. Bacterial DNA was recovered from 253 samples, with 250 samples yielding both plant and bacterial DNA sequences. Across all samples, there were 2,214,404 reads of plant DNA, assigned to 2,760 unique ZOTUs (zero-radius operational taxonomic units; unique DNA sequences [81]) in 124 families. For bacteria, 2,449,012 reads were assigned to 3,226 unique ZOTUs in 194 families. Almost all plant and most bacterial DNA could be assigned to family (97.8% and 76.8% of ZOTUs, 95.5% and 87.2% of reads, respectively). Somewhat less plant and bacterial DNA could be assigned to genus (85.3% and 49.1% of ZOTU and 86.9% and 71.6% of reads, respectively).

We primarily focused on the extent to which honey bee niche breadth varied over latitude. As additional potential sources of variation in observed niche breadth, we also recorded year of sampling, number of hives from which the honey was pooled for the sample, number of beekeepers from which the honey was pooled for the sample, hive type, method of honey extraction, country, and longitude. However, all of these other covariates were significantly confounded with latitude (Appendix *S2*), largely due to the clustering of similar values of the predictors to particular latitudes. As this confounding presented problems of identifiability in models including latitude and other predictors, we first tested for trends with respect to latitude alone, then examined whether these additional predictors could account for residual variation in the data. In addition, sequencing depth may affect the detectability of interaction partners. Indeed, the number of plants or bacteria recovered from a sample was correlated with the total number of reads obtained from the sample in question (Appendix *S3*). We therefore focused on overall trends in niche breadth with respect to latitude, identified using quasi-Poisson regression models of group-specific richness to the logarithm of total reads and absolute latitude. After examining the overall trends, we tested whether these trends varied with year of sampling, number of hives, hive type, and method of harvest. These other factors did not have major effects on trends in niche breadth over latitude, except where samples with a certain factor value were clustered in a narrow range of low latitudes (Appendix *S4*). This concerned in particular the top-bar hives and the hives from which honey was extracted by squeezing. We examined trends in honey bee niche breadths measured using plant and bacterial ZOTUs, genera, and families. Since the results were largely consistent across taxonomic levels for both kingdoms, we present results for ZOTUs in the main text and results for genera and families in Appendix *S5*. To account for the possibility that over-representation of some countries in our dataset could influence our results, we conducted a rarefaction analysis of the models relating niche breadth to absolute latitude and log(reads). This analysis showed very little effect of geographically-aggregated sampling on our conclusions (Appendix *S6*). Separately, to assess whether the trends we find in the sampling around the world are consistent within the native range of *Apis mellifera* (Africa, Middle East and most of Europe, following Requier et al. [25, 27]), we fit an additional round of models relating niche breadth to absolute latitude and log(reads) using only the samples collected within this range. This analyses confirmed all the trends obtained when using the full dataset (Appendix *S7*).

### Does the niche breadth of the honey bee vary over latitude in terms of plant and bacterial interaction partners?

**Fig. 2:**
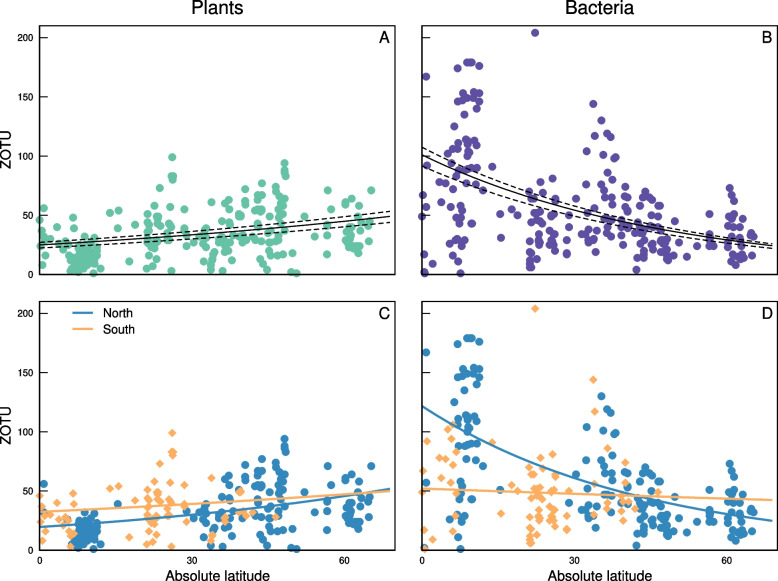
The niche breadth of the honey bee varied significantly over latitude, though the trends differed depending on whether we define niche breadth using the number of plant (**A**, **C**) or bacteria (**B**, **D**) ZOTUS identified in a honey sample. **A** Honey bees visited more plant ZOTUs towards the poles (i.e., broader niches), and **C** the strength of this trend was similar in the Northern (blue circles) and Southern (orange diamonds) hemispheres. **B** Honey bees encountered fewer bacterial ZOTUs towards the poles (i.e., narrower niches), though this trend was much weaker (but still significant) in the Southern hemisphere. The curves represent fitted values from a model including the log of the total number of reads to account for increasing detectability with increasing sequence yield. The solid line represents the fit for the mean number of reads for each taxonomic group and the dotted lines represent the 25% and 75% quantiles. For the mean number of reads, we also show results for each hemisphere separately

Niche breadth defined as number of plant ZOTUs per honey sample increased significantly with increasing latitude (Fig. 2A; Table 1), while niche breadth based on bacterial ZOTUs decreased significantly with increasing latitude (and was thereby highest in the tropics; Fig. 2B). For both kingdoms, these trends were significant in the Northern and Southern hemispheres, though the trend was much weaker for bacteria in the Southern hemisphere (Fig. 2C-D; Table 2). This appears to be due to particularly high niche breadth just north of the Equator, whereas we have few samples from equivalent latitudes in the southern hemisphere.

**Table 1:** Results for quasi-Poisson regressions of ZOTU per honey sample against absolute latitude and the log of total number of reads

	Absolute latitude			log(Reads)		
Group	$\varvec{\beta }$	*F*	*p*	$\varvec{\beta }$	*F*	*p*
Plants	$\varvec{9.78\times 10}^{\varvec{-3}}$	**42.8**	$\varvec{<}$**0.001**	$\varvec{3.03\times 10}^{\varvec{-1}}$	**22.7**	$\varvec{<}$**0.001**
Bacteria	$\varvec{-2.07\times 10}^{\varvec{-2}}$	**61.3**	$\varvec{<}$**0.001**	$\varvec{2.31\times 10}^{\varvec{-1}}$	**7.00**	**0.009**

Shown are coefficients, *F*-statistics, and *p*-values for absolute latitude and log(total reads). Regarding coefficients, note that in a quasi-Poisson regression, $y=e^{intercept + \beta }$. For genera and families, see Appendix *S5*, Tables *S10-11*

**Table 2:** Results for quasi-Poisson regressions of ZOTU per honey sample aganst absolute latitude (abs(lat)), log(total reads), hemisphere, and the interaction between latitude and hemisphere

	Plants			Bacteria		
	df	F	P	df	F	P
abs(lat)	1, 246	**849**	$\varvec{<}$**0.001**	1, 248	**322**	$\varvec{<}$**0.001**
log(reads)	2, 246	**19.9**	$\varvec{<}$**0.001**	2, 248	**10.5**	**0.001**
Hemisphere	1, 246	**52.1**	$\varvec{<}$**0.001**	1, 248	**223**	$\varvec{<}$**0.001**
abs(lat):Hemisphere	1, 246	0.803	0.371	1, 248	**6.21**	**0.013**

Shown are coefficients, degrees of freedom, *F*-statistics, and *p*-values. Significant terms (${<}$0.005) are indicated in **bold**

### Do latitudinal trends vary among bacterial groups of known impacts?

**Fig. 3:**
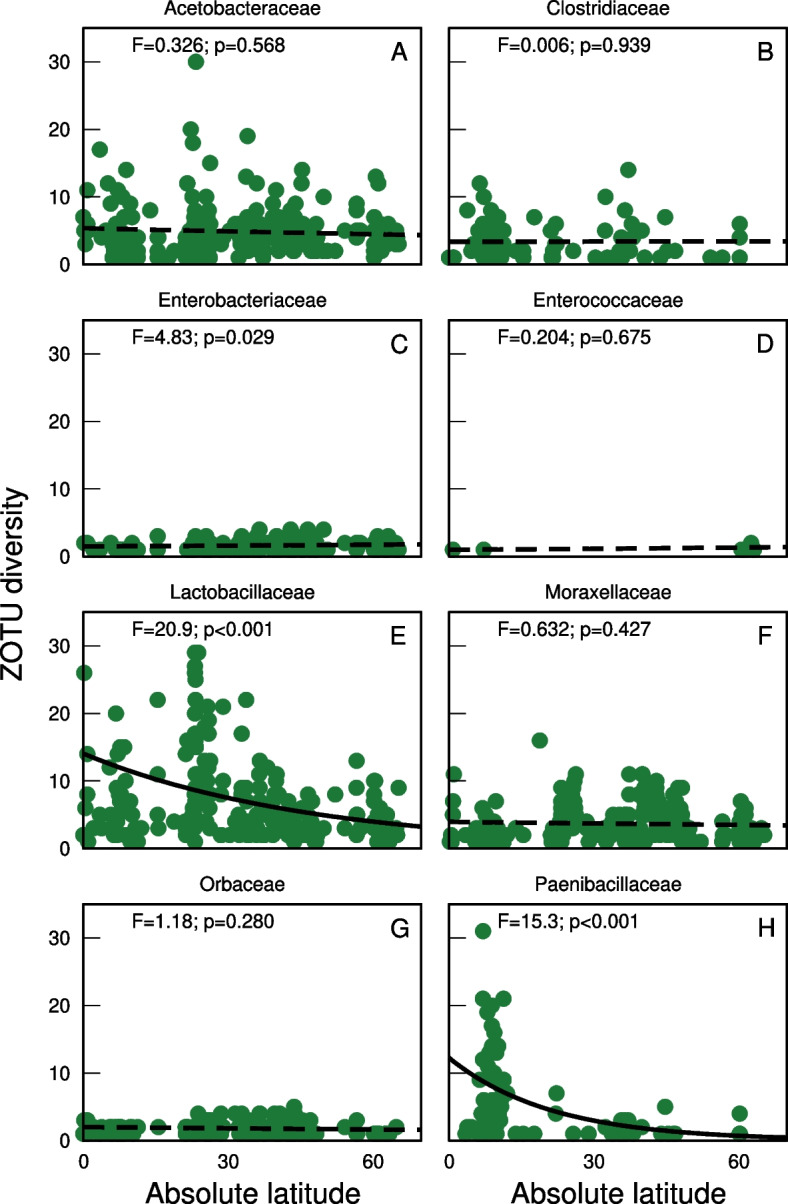
Latitudinal trends in the richness of focal bacterial groups encountered by honey bees. Shown are the number of ZOTUs recovered per honey sample for bacterial families (coloured circles). Black lines indicate fits of models relating ZOTU richness in the focal family to absolute latitude and the log number of reads in the sample. For simplicity, we show results for the mean number of reads per sample, across all samples which included the focal family). Dashed lines indicate non-significant trends. For the effect of absolute latitude in each model, we provide pseudo-*F* and *p*-values as insets. These values were derived from anova tests

The number of Paenibacillaceae and Lactobacillaceae ZOTUs in honey declined significantly with increasing latitude, while the richness of other focal families did not show any clear latitudinal trends (Fig. 3, Table 3). This suggests that latitudinal gradients in bacteria richness vary between taxonomic groups.

**Table 3:** Results for quasi-Poisson regressions of ZOTUs within selected bacterial families against log(total reads, absolute latitude, hemisphere (categorical), and their interaction

	Absolute latitude			log(Reads)		
Taxon	$\varvec{\beta }$	*F*	*p*	$\varvec{\beta }$	*F*	*p*
Acetobacteraceae	−0.00303	0.326	0.568	0.191	3.43	0.065
Clostridiaceae	0.000193	0.006	0.939	0.126	0.258	0.613
Enterobacteriaceae	**0.00265**	**4.83**	**0.0295**	**0.370**	**14.5**	$\varvec{<}$**0.001**
Enterococcaceae	0.00488	0.204	0.675	**−1.43**	**14.0**	**0.020**
Lactobacillaceae	**−0.0210**	**20.9**	$\varvec{<}$**0.001**	**0.597**	**14.7**	$\varvec{<}$**0.001**
Moraxellaceae	−0.00204	0.632	0.427	**0.639**	**27.0**	$\varvec{<}$**0.001**
Orbaceae	−0.00338	1.18	0.280	0.0434	0.185	0.668
Paenibacillaceae	**−0.0474**	**15.3**	$\varvec{<}$**0.001**	0.606	3.19	0.078

Shown are coefficients, *F*-statistics, and *p*-values. Regarding coefficients, note that in a quasi-Poisson regression, $y=e^{intercept + \beta }$. Significant trends (${<}$0.05) are indicated in **bold**

## Discussion

The Latitudinal Biotic Interaction Hypothesis predicts a general increase in the strength of biotic interactions and a corresponding decrease in niche breadth towards the Equator [10]. When exploring this pattern using the honey bee *Apis mellifera* as a model species, we found different patterns in associations with different kingdoms. Honey bees visited more plants at higher latitudes (i.e., a wider niche), but were associated with more bacteria in the tropics. In both cases, our results could reflect differences in foraging behaviour among honey bee subspecies [23] as bee subspecies have different ranges [27]. It is also possible that there are differences in niche breadth patterns between the native and introduced range of *A. mellifera*, although we found the same results for samples collected within the native range and our full dataset (Appendix *S7*). Identifying the subspecies in each hive and the extent to which their foraging may be affected by adaptation to local plants [27] would be interesting directions for future research, but do not provide a ready explanation for the differences we observe between numbers of plants and bacteria represented in honey. These contrasting trends are instead likely to reflect differences in actively-chosen vs. chance interactions.

### Honey bees’ plant usage aligns with the Latitudinal Biotic Interaction Hypothesis

In our global dataset, more plant ZOTUs and genera were found in honey samples at higher latitudes. The observed increase in number of taxa was modest, but significant, with approximately 3.5 additional plant ZOTUs or one additional plant genus visited when moving 10 of absolute latitude towards the poles (Table 1; assuming the mean number of reads per sample). The observed increase in number of families was even smaller, and may be due to the spatial aggregation of our data (see Appendix *S6*). As honey bees select individual plants to visit rather than higher-order taxa, it is not surprising that trends in niche breadth based on plants are strongest at the level of ZOTUs and weakest for families. Although we do not test the strength of plant-honey bee interactions directly, this increase in niche breadth towards the poles is consistent with the Latitudinal Biotic Interaction Hypothesis, which predicts fewer but stronger interactions in the tropics [10]. The observed pattern is also consistent with reports of plants having fewer flower-visitors towards the Equator [15], but it contrasts with a recent global review which suggested tropical pollinators tend to be more generalist [20]. This disparity, however, is likely due to confounding changes in the composition of the pollinator fauna with changes in latitude. Towards the tropics, we see an increasing representation of vertebrates and social insects among pollinators [20]. Overall, our findings underline the importance of making a consistent, apples-to-apples comparison of niche breadth over latitude.

The widening range of plant usage towards the poles contrasts markedly with the expectation from Neutral Theory. If honey bees interacted with a constant, random subset of all plants available, then we would expect higher number of interaction partners where flowering plant richness is higher [8, 82]. Under this scenario, we would expect broader niches in the tropics rather than the narrower niches empirically observed.

Arguably, our finding of increasing specialisation towards the tropics is actually based on a conservative test. First, the plant interaction partners of honey bees will include some proportion of passively-attracted interaction partners. Honey bees will encounter some wind-dispersed pollen in the environment [46, 83], adding noise to the data on presumptively actively-attracted interaction partners. Second, the latitudinal trend in flowering plant richness [5, 7, 84] is not universal. The overall pattern is disrupted by low richness in tropical deserts and high richness in temperate hotspots, such as the South African Cape region [85] and the temperate forest in southern Chile [86]. Thus, finding a consistent trend of widening niches towards higher latitudes *despite* variation in the underlying pattern of plant richness, and some noise from wind-pollinated taxa, does attest to a strong ecological signal of latitude.

An important factor contributing to the number of plant species visited at any latitude is honey bee behaviour. Honey bees select flowers both individually and as a colony, and use communication among foragers to share information about available resources [87]. Foragers scout the area around the hive and inform the colony of the best nectar sources, with a preference for those closer to the hive [34]. They then tend to prefer plants with higher nectar volume and higher sugar content of the nectar [88]. If a few nearby plant taxa provide abundant, high-sugar nectar, the overall richness of flowering plant taxa may have little impact on honey bee interactions. This choice to focus the nectar foraging on a single or few abundant plant taxa may be caused both by native, wild plants or crop plants being abundant in the proximity of the hive [89]. Large fields of nectar-producing crop plants, such as oilseed rape or sunflower, are known to attract honey bees and guide their foraging towards these crops, although honey bees’ preferences for these crops depends on the other plants available [90, 91]. In a similar manner, abundantly-flowering wild plants such as raspberry or willow can be the main nectar source for honey bees [29, 83]. If large crop fields or highly-abundant wild plants occur more often towards the tropics, this could explain the decrease in interaction partners towards equator. However, we are not aware of any evidence for such a pattern. In this context, vegetation mapping in the vicinity of the hives from which the honey samples originate would be an extremely useful addition to future studies. This would allow for a direct comparison of the availability of flowers to the flowers chosen by honey bees, across latitudes.

As well as local plant abundance, honey bee niche breadths will depend on local flowering periods. Broader niches at higher latitudes could result from shorter flowering periods if honey bees at high latitudes need to switch plants more often through the season than do tropical honey bees. While in general plant flowering and bee foraging are restricted to a few months at high latitudes, *peak flower abundance* for different species is likely to be on different days or weeks. Following the logic of floral abundance outlined above, this could result in rapid switching between different plant species to take advantage of peak nectar availability. In the tropics, where temperature is less restrictive for both flowering and foraging, some plants may flower for a much greater proportion of the year and offer honey bees a reliable long-term resource. We are not aware of any global comparison of flowering times across latitudes, but such an analysis would offer a key contribution to understanding how local plant richness translates to short-term resource availability for pollinators.

Longer honey storage within the hive could also contribute to the trends we observe, if honey bees select similar numbers of the best-quality resources at any given time regardless of latitude, these resources have higher temporal turnover at higher latitudes, and honey samples from high-latitude hives include honey stored over a longer period than honey in tropical hives which may be harvested multiple times per year. This possibility is contradicted by recent research from high latitudes (specifically, Finland) which reveals a turnover of plant composition within stored honey over approximately two months [29, 30]. Turnover in plant composition within honey reflects ongoing consumption of honey by the bees themselves and means that a honey sample collected at the end of the active foraging season represents only flowering plants used within the last one to two months. Thus, although honey may be harvested by humans multiple times per year at low latitudes and only once at high latitudes, honey bees themselves create a standardised sampling window by their own consumption of honey, making longer honey storage an unlikely cause of the trends we observe.

### Honey bees’ encounters with bacteria is consistent with Neutral Theory, assuming bacterial richness increases towards the equator

The richness of bacterial taxa in honey samples increased strongly towards the equator. This pattern is consistent with our prediction of more diverse honey bee-microbe associations at lower latitudes, based on the assumption that bacteria are more species-rich in the tropics. As honey bees meet most of the bacteria they are associated with through chance encounters in the environment [92], we expected that the number of microbes encountered should reflect microbial richness in the environment [8, 82].

It is important to reiterate that global trends in bacterial richness are not yet well-documented and preliminary studies show conflicting results ([93] but see e.g. [94]). As with plants, we did not empirically sample bacterial species richness in the environment. Instead, we base our assumption about bacterial richness on trends in other taxonomic groups, which are generally more species-rich at lower latitudes [5]. There is no obvious *a priori* reason why richness in micro- and macro-organisms should show different trends, but we eagerly await stronger empirical evidence of global bacteria diversity patterns.

If bacteria are indeed more species-rich in the tropics, our current results are consistent with Neutral Theory [8, 82]. As honey bees encounter most of their associated bacteria randomly in the environment, we would expect them to have more associated bacteria where the local bacteria community is richer. If our underlying assumption is false and bacterial richness is constant over latitude or higher towards the poles, this implies that honey bees are more generalist in regard to associations with bacteria in the tropics for other reasons. However, greater generalism in the tropics would also be at odds with the Latitudinal Biotic Interaction Hypothesis, which generally predicts fewer but stronger interactions in the tropics. To find support to either hypothesis, and to explore the patterns more in detail, more research on global bacterial diversity trends, as well as the local bacterial diversity a particular honey bee colony can potentially associate with, is needed.

Despite the clear overall trend, we observed substantial variation between focal families of bacteria (Fig. 3). As examples of functionally-important groups, we specifically examined eight families. Seven of these families have known members with strong associations with honey bees which range from pathogens to symbionts living in bee guts or hives [39, 73, 80]. The eighth group (family Clostridiaceae) includes an environmental bacterium *Clostridium botulinum* pathogenic to animals, transported by honey bees and commonly found in honey [75]. Among these groups, we found pronounced differences in latitudinal pattern. The number of Paenibacillaceae or Lactobacillaceae ZOTUs in honey increased towards equator, whereas the other families showed constant ZOTU richness across latitudes.

Among the Lactobacillaceae, two species are known to occur in the guts of all honey bees [37, 95]. The ZOTU richness within these species is therefore unlikely to vary across latitudes. Instead, the increase in richness towards the tropics is likely due to greater richness of other lactic acid bacteria living in nectar [39, 41, 96, 97] or elsewhere in the environment [98]. The Paenibacillaceae, meanwhile, include bee pathogens (e.g.*Paenibacillus larvae* and *P. alvei*) as well as species producing antimicrobials and insecticides, offering protection against insect herbivores and pathogens (e.g. against the pathogen *Clostridium botulinum* [99, 100]. As these and the other focal families we consider have such a wide range of functions, it is possible that there are various drivers of richness trends within, as well as between, bacterial families. Unravelling these trends is beyond the scope of the present study but offers a broad field for further studies.

## Conclusions

Focusing on the honey bee allowed us to apply a consistent methodology for measuring changes in the richness of associations with other taxa (niche breadth) over latitude. Our main finding was that latitudinal trends in niche breadth varied with the type of interaction. While the Latitudinal Biotic Interaction Hypothesis posits a general increase in the strength of biotic interactions towards the Equator [10], our study suggests that this is most likely true specifically for *actively selected* interaction partners; i.e., the plants that the honey bees choose to visit. For such interactions, the increase in the number of interaction partners is consistent with the Latitudinal Biotic Interaction Hypothesis, which predicts fewer (but stronger; not tested here) interactions in the tropics. However, for interactions resulting from chance encounters in the environment, latitudinal pattern in interaction richness was completely different. In this case, our results fail to reject the Neutral Theory, assuming bacterial richness increases towards the equator [82, 101]. Since all our findings relate to a single species with the same fundamental niche across the globe, our study is the first to show how different ecological theories may apply to different types of interactions. With this, the stage is set for extended assessments of global patterns in interaction richness over latitudes.

## Supplementary Information

Supplementary Material 1.

## Data Availability

Polished datasets and R scripts used in all analyses are available here https://doi.org/10.5281/zenodo.14914453. Raw datasets used in this study are available here https://www.ncbi.nlm.nih.gov/bioproject/PRJNA1137582 and here https://www.ncbi.nlm.nih.gov/bioproject/PRJNA1152939 (except samples ITpSUO191-94 and 16SSUO191-94, which are not part of this study). DNA sequences used in this study were deposited to Sequence Read Archive repository, available in the BioProject PRJNA1152939 (https://www.ncbi.nlm.nih.gov/sra/PRJNA1152939) and in PRJNA1137582 (https://www.ncbi.nlm.nih.gov/sra/PRJNA1137582, without samples ITpSUO191-94 and 16SSUO191-94).

## References

[1] Elton C. Anim Ecol. London: Sidgwick & Jackson, Ltd.; 1927.

[2] Poulin R. Are there general laws in parasite ecology? Parasitology. 2007;134(6):763–76. 10.1017/S0031182006002150. 17234043 10.1017/S0031182006002150

[3] Begon M, Harper JL, Townsend CR. Ecology. Individuals, populations and communities. Oxford: Blackwell; 1986.

[4] Rohde K. Latitudinal gradients in species diversity: the search for the primary cause. Oikos. 1992;65(3):514–27.

[5] Gillman LN, Wright SD, Cusens J, Mcbride PD, Malhi Y, Whittaker RJ. Latitude, productivity and species richness. Global Ecol Biogeo. 2015;24(1):107–17. 10.1111/GEB.12245/SUPPINFO.

[6] Cusens J, Wright SD, McBride PD, Gillman LN. What is the form of the productivity-animal-species-richness relationship? A critical review and meta-analysis. Ecology. 2012;93(10):2241–52. 10.1890/11-1861.1. 23185885 10.1890/11-1861.1

[7] Brummitt N, Araújo AC, Harris T. Areas of plant diversity-What do we know? Plants People Planet. 2021;3(1):33–44. 10.1002/ppp3.10110.

[8] Hubbell SP. Neutral theory and the evolution of ecological equivalence. Ecology. 2006;87(6):1387–98. 10.1890/0012-9658(2006)87[1387:NTATEO]2.0.CO;2. 16869413 10.1890/0012-9658(2006)87[1387:ntateo]2.0.co;2

[9] Vázquez DP, Stevens RD. The latitudinal gradient in niche breadth: concepts and evidence. Am Nat. 2004;164(1):1–19. 10.1086/421445. 15266376 10.1086/421445

[10] Schemske DW, Mittelbach GG, Cornell HV, Sobel JM, Roy K. Is There a Latitudinal Gradient in the Importance of Biotic Interactions? Ann Rev Ecol Evol Sys. 2009;2009(40):245–69. 10.1146/annurev.ecolsys.39.110707.173430.

[11] Saupe EE, Myers CE, Peterson AT, Soberón J, Singarayer J, Valdes P, et al. Non-random latitudinal gradients in range size and niche breadth predicted by spatial patterns of climate. Global Ecol Biogeo. 2019;28(7):928–42. 10.1111/geb.12904.

[12] McKinnon L, Smith PA, Nol E, Martin JL, Doyle FI, Abraham KF, et al. Lower predation risk for migratory birds at high latitudes. Science. 2010;327:326–7. 10.1126/science.1183010. 20075251 10.1126/science.1183010

[13] Roslin T, Hardwick B, Novotny V, Petry WK, Andrew NR, Asmus A, et al. Higher predation risk for insect prey at low latitudes and elevations. Science. 2017;356:742–4. 10.1126/science.aaj1631. 28522532 10.1126/science.aaj1631

[14] Zvereva EL, Kozlov MV. Latitudinal gradient in the intensity of biotic interactions in terrestrial ecosystems: sources of variation and differences from the diversity gradient revealed by meta-analysis. Ecol Lett. 2021;24:2506–20. 10.1111/ele.13851. 34322961 10.1111/ele.13851

[15] Olesen JM, Jordano P. Geographic patterns in plant-pollinator mutualistic networks. Ecology. 2002;83(9):2416–24. 10.1890/0012-9658(2002)083[2416:GPIPPM]2.0.CO;2.

[16] Moles AT, Bonser SP, Poore AGB, Wallis IR, Foley WJ. Assessing the evidence for latitudinal gradients in plant defence and herbivory. Funct Ecol. 2011;25:380–8. 10.1111/j.1365-2435.2010.01814.x.

[17] Mottl O, Fibich P, Klimes P, Volf M, Tropek R, Anderson-Teixeira K, et al. Spatial covariance of herbivorous and predatory guilds of forest canopy arthropods along a latitudinal gradient. Ecol Lett. 2020;23:1499–510. 10.1111/ele.13579. 32808457 10.1111/ele.13579

[18] Cirtwill AR, Stouffer DB, Romanuk TN. Latitudinal gradients in biotic niche breadth vary across ecosystem types. Proc R Soc B. 1819;2015(282):20151589. 10.1098/rspb.2015.1589. 10.1098/rspb.2015.1589 PMC4685804 26559955

[19] Ollerton J, Cranmer L, Science E, Campus P. Latitudinal trends in plant-pollinator interactions: are tropical plants more specialised? Oikos. 2002;98(2):340–50. 10.1034/j.1600-0706.2002.980215.x.

[20] Schleuning M, Fründ J, Klein AM, Abrahamczyk S, Alarcón R, Albrecht M, et al. Specialization of mutualistic interaction networks decreases toward tropical latitudes. Curr Biol. 2012;22(20):1925–31. 10.1016/j.cub.2012.08.015. 22981771 10.1016/j.cub.2012.08.015

[21] Anstett DN, Nunes KA, Baskett C, Kotanen PM. Sources of controversy surrounding latitudinal patterns in herbivory and defense. Trends Ecol Evol. 2016;31:789–802. 10.1016/j.tree.2016.07.011. 27545844 10.1016/j.tree.2016.07.011

[22] Morris RJ, Gripenberg S, Lewis OT, Roslin T. Antagonistic interaction networks are structured independently of latitude and host guild. Ecol Lett. 2014;17(3):340–9. 10.1111/ele.12235. 24354432 10.1111/ele.12235 PMC4262010

[23] Crane E. Bees and beekeeping: science, practice and world resources. Oxford: Heineman Newnes; 1990.

[24] Whitfield CW, Behura SK, Berlocher SH, Clark AG, Johnston JS, Sheppard WS, et al. Thrice Out of Africa: Ancient and Recent Expansions of the Honey Bee. Apis mellifera Science. 2006;314(October):642–5. 10.1126/science.1132772. 17068261 10.1126/science.1132772

[25] Tihelka E, Cai C, Pisani D, Donoghue PCJ. Mitochondrial genomes illuminate the evolutionary history of the Western honey bee (*Apis mellifera*). Sci Rep. 2020;10. 10.1038/s41598-020-71393-0. 10.1038/s41598-020-71393-0 PMC7471700 32884034

[26] GBIF.org. GBIF Occurrence Download. 2024. 10.15468/dl.q4mnfd.

[27] Requier F, Garnery L, Kohl P, Njovu H, Pirk C, Crewe R, et al. The Conservation of Native Honey Bees Is Crucial. Trends Ecol Evol. 2019;34:789–98. 10.1016/j.tree.2019.04.008. 31072605 10.1016/j.tree.2019.04.008

[28] Meixner MD, Pinto MA, Bouga M, Kryger P, Ivanova E, Fuchs S. Standard methods for characterising subspecies and ecotypes of *Apis mellifera*. J Apicult Res. 2013;52(4). 10.3896/IBRA.1.52.4.05.

[29] Leponiemi M, Freitak D, Moreno-Torres M, Pferschy-Wenzig EM, Becker-Scarpitta A, Tiusanen M, et al. Honeybees’ foraging choices for nectar and pollen revealed by DNA metabarcoding. Sci Rep. 2023;13. 10.1038/s41598-023-42102-4. 10.1038/s41598-023-42102-4 PMC10484984 37679501

[30] Wirta H, Jones M, Peña-Aguilera P, Chacón-Duque C, Vesterinen E, Ovaskainen O, et al. The role of seasonality in shaping the interactions of honeybees with other taxa. Ecol Evol. 2023;13. 10.1002/ECE3.10580. 10.1002/ece3.10580 PMC10560870 37818248

[31] FAO Food and Agriculture Organisation of the United Nations. FAOSTAT. 2021. https://www.fao.org/faostat/en/#home. Accessed 27 Oct 2021.

[32] Klein AM, Vaissière BE, Cane JH, Steffan-Dewenter I, Cunningham SA, Kremen C, et al. Importance of pollinators in changing landscapes for world crops. Proc R Soc B. 2007;274(1608):303–13. 10.1098/rspb.2006.3721. 17164193 10.1098/rspb.2006.3721 PMC1702377

[33] Hendriksma HP, Shafir S. Honey bee foragers balance colony nutritional deficiencies. Behav Ecol Sociobiol. 2016;70(4):509–17. 10.1007/s00265-016-2067-5.

[34] Seeley TD, Camazine S, Sneyd J. Behavioral Ecology and Sociobiology Collective decision-making in honey bees: how colonies choose among nectar sources. Behav Ecol Sociobiol. 1991;28:277–90.

[35] Brodschneider R, Gratzer K, Kalcher-Sommersguter E, Heigl H, Auer W, Moosbeckhofer R, et al. A citizen science supported study on seasonal diversity and monoflorality of pollen collected by honey bees in Austria. Sci Rep. 2019;9:16633. 10.1038/s41598-019-53016-5. 31719621 10.1038/s41598-019-53016-5 PMC6851371

[36] Engel P, James RR, Koga R, Kwong WK, McFrederick QS, Moran NA. Standard methods for research on *Apis mellifera* gut symbionts. J Apicult Res. 2015;52(4):1–24. 10.3896/IBRA.1.52.4.07.

[37] Moran NA, Sloan DB. The Hologenome Concept: Helpful or Hollow? PLoS Biol. 2015;13(12):1–10. 10.1371/journal.pbio.1002311. 10.1371/journal.pbio.1002311 PMC4670207 26636661

[38] Kešnerová L, Moritz R, Engel P. *Bartonella apis* sp. nov., a honey bee gut symbiont of the class Alphaproteobacteria. Int J Sys Evol Microbiol. 2016;66:414–21. 10.1099/IJSEM.0.000736. 10.1099/ijsem.0.000736 26537852

[39] Raymann K, Moran NA. The role of the gut microbiome in health and disease of adult honey bee workers. Curr Op Insect Sci. 2018;26:97–104. 10.1016/j.cois.2018.02.012. 10.1016/j.cois.2018.02.012 PMC6010230 29764668

[40] Aizenberg-Gershtein Y, Izhaki I, Halpern M. Do Honeybees Shape the Bacterial Community Composition in Floral Nectar? PLoS ONE. 2013;8:e67556. 10.1371/journal.pone.0067556. 23844027 10.1371/journal.pone.0067556 PMC3701072

[41] Hietaranta E, Juottonen H, Kytöviita MM. Honeybees affect floral microbiome composition in a central food source for wild pollinators in boreal ecosystems. Oecologia. 2023;201:59–72. 10.1007/s00442-022-05285-7. 36434466 10.1007/s00442-022-05285-7 PMC9813210

[42] de Oliveira Scoaris D, Hughes FM, Silveira MA, Evans JD, Pettis JS, Bastos EMAF, et al. Microbial communities associated with honey bees in Brazil and in the United States. Braz J Microbiol. 2021;52(4):2097–115. 10.1007/s42770-021-00539-7. 34264502 10.1007/s42770-021-00539-7 PMC8578358

[43] Snowdon JA, Cliver DO. Microorganisms in honey. Int J Food Microbiol. 1996;31:1–26. 10.1016/0168-1605(96)00970-1. 8880294 10.1016/0168-1605(96)00970-1

[44] Fürst MA, McMahon DP, Osborne JL, Paxton RJ, Brown MJF. Disease associations between honeybees and bumblebees as a threat to wild pollinators. Nature. 2014;506(7488):364–6. 10.1038/NATURE12977. 24553241 10.1038/nature12977 PMC3985068

[45] Vannette RL. The Floral Microbiome: Plant, Pollinator, and Microbial Perspectives. Ann Rev Ecol Evol Sys. 2020;51:363–86. 10.1146/ANNUREV-ECOLSYS-011720-013401.

[46] Wirta H, Bahram M, Miller K, Roslin T, Vesterinen E. Reconstructing the ecosystem context of a species: Honey-borne DNA reveals the roles of the honeybee. PLoS ONE. 2022;17(7). 10.1371/journal.pone.0268250. 10.1371/journal.pone.0268250 PMC9278776 35830374

[47] Bovo S, Ribani A, Utzeri VJ, Schiavo G, Bertolini F, Fontanesi L. Shotgun metagenomics of honey DNA: Evaluation of a methodological approach to describe a multi-kingdom honey bee derived environmental DNA signature. PLoS ONE. 2018;13:e0205575. 10.1371/journal.pone.0205575. 30379893 10.1371/journal.pone.0205575 PMC6209200

[48] Bovo S, Utzeri VJ, Ribani A, Cabbri R, Fontanesi L. Shotgun sequencing of honey DNA can describe honey bee derived environmental signatures and the honey bee hologenome complexity. Sci Rep. 2020;10(1). 10.1038/s41598-020-66127-1. 10.1038/s41598-020-66127-1 PMC7283317 32518251

[49] Qian H, Zhang J, Jiang M. Global patterns of taxonomic and phylogenetic diversity of flowering plants: Biodiversity hotspots and coldspots. Plant Div. 2023;45:265–71. 10.1016/j.pld.2023.01.009. 10.1016/j.pld.2023.01.009 PMC10311147 37397596

[50] Gillman LN, Wright SD, Cusens J, McBride PD, Malhi Y, Whittaker RJ. Latitude, productivity and species richness. Glob Ecol Biogeo. 2014. 10.1111/geb.12245.

[51] Tian J, He N, Hale L, Niu S, Yu G, Liu Y, et al. Soil organic matter availability and climate drive latitudinal patterns in bacterial diversity from tropical to cold temperate forests. Funct Ecol. 2018;32(1):61–70.

[52] Hendershot JN, Read QD, Henning JA, Sanders NJ, Classen AT. Consistently inconsistent drivers of microbial diversity and abundance at macroecological scales. Ecology. 2017;98:1757–63. 10.1002/ecy.1829. 28380683 10.1002/ecy.1829

[53] Delgado-Baquerizo M, Maestre FT, Reich PB, Jeffries TC, Gaitan JJ, Encinar D, et al. Microbial diversity drives multifunctionality in terrestrial ecosystems. Nature. 2016;7:10541. 10.1038/ncomms10541. 10.1038/ncomms10541 PMC4738359 26817514

[54] Tedersoo L, Bahram M, Põlme S, Kõljalg U, Yorou NS, Wijesundera R, et al. Global diversity and geography of soil fungi. Science. 2014;346. 10.1126/science.1256688. 10.1126/science.1256688 25430773

[55] Bahram M, Hildebrand F, Forslund SK, Anderson JL, Soudzilovskaia NA, Bodegom PM, et al. Structure and function of the global topsoil microbiome. Nature. 2018;560(7717):233–7. 30069051 10.1038/s41586-018-0386-6

[56] Abrego N, Furneaux B, Hardwick B, Somervuo P, Palorinne I, Aguilar-Trigueros CA, et al. Airborne DNA reveals predictable spatial and seasonal dynamics of fungi. Nature. 2024;631:835–42. 10.1038/s41586-024-07658-9. 38987593 10.1038/s41586-024-07658-9 PMC11269176

[57] Ladau J, Sharpton TJ, Finucane MM, Jospin G, Kembel SW, O’Dwyer J, et al. Global marine bacterial diversity peaks at high latitudes in winter. ISME J. 2013;7:1669–77. 10.1038/ismej.2013.37. 23514781 10.1038/ismej.2013.37 PMC3749493

[58] Andam CP, Doroghazi JR, Campbell AN, Kelly PJ, Choudoir MJ, Buckley DH. A latitudinal diversity gradient in terrestrial bacteria of the genus *Streptomyces*. mBio. 2016;7:e02200–15. 10.1128/mBio.02200-15. 10.1128/mBio.02200-15 PMC4817263 27073097

[59] Wang Z, Jiang Y, Zhang M, Chu C, Chen Y, Fang S, et al. Diversity and biogeography of plant phyllosphere bacteria are governed by latitude-dependent mechanisms. New Phy. 2023;240:1534–47. 10.1111/nph.19235. 10.1111/nph.19235 37649282

[60] Chen S, Yao H, Han J, Chang L, Song J, Shi L, et al. Validation of the ITS2 region as a novel DNA barcode for identifying medicinal plant species. PLoS ONE. 2010;5:1–8. 10.1371/journal.pone.0008613. 10.1371/journal.pone.0008613 PMC2799520 20062805

[61] White T, Bruns T, Lee J, Taylor M. Amplification and direct sequencing of fungal ribosomal RNA genes for phylogenetics. In: PCR protocols: a guide to methods and applications. Academic Press, London, UK; 1990. pp. 315–22.

[62] Walters W, Hyde ER, Berg-Lyons D, Ackermann G, Humphrey G, Parada A, et al. Improved Bacterial 16S rRNA Gene (V4 and V4-5) and Fungal Internal Transcribed Spacer Marker Gene Primers for Microbial Community Surveys crossmark. mSystems. 2016;1. 10.1128/mSystems.00009-15. 10.1128/mSystems.00009-15 PMC5069754 27822518

[63] Caporaso JG, Lauber CL, Walters WA, Berg-Lyons D, Lozupone CA, Turnbaugh PJ, et al. Global patterns of 16S rRNA diversity at a depth of millions of sequences per sample. Proc Natl Acad Sci U S A. 2011;108:4516–22. 10.1073/pnas.1000080107. 20534432 10.1073/pnas.1000080107 PMC3063599

[64] Vesterinen EJ, Puisto AIE, Blomberg AS, Lilley TM. Table for five, please: dietary partitioning in boreal bats. Ecol Evol. 2018;8(22):10914–37. 10.1002/ece3.4559. 30519417 10.1002/ece3.4559 PMC6262732

[65] Kaunisto KM, Roslin T, Forbes MR, Morrill A, Sääksjärvi IE, Puisto AIE, et al. Threats from the air: Damselfly predation on diverse prey taxa. J Anim Ecol. 2020;89:1365–74. 10.1111/1365-2656.13184. 32124439 10.1111/1365-2656.13184

[66] Rognes T, Flouri T, Nichols B, Quince C, Mahé F. VSEARCH: a versatile open source tool for metagenomics. PeerJ. 2016;e2584. 10.7717/peerj.2584. 10.7717/peerj.2584 PMC5075697 27781170

[67] Cole JR, Wang Q, Cardenas E, Fish J, Chai B, Farris RJ, et al. The Ribosomal Database Project: improved alignments and new tools for rRNA analysis. Nucleic Acids Res. 2008;37:D141–5. 10.1093/nar/gkn879. 19004872 10.1093/nar/gkn879 PMC2686447

[68] Banchi E, Ametrano CG, Greco S, Stanković D, Muggia L, Pallavicini A. PLANiTS: a curated sequence reference dataset for plant ITS DNA metabarcoding. Database. 2020;2020:baz155. 10.1093/database/baz155. 32016319 10.1093/database/baz155 PMC6997939

[69] Edgar RC. SINTAX: a simple non-Bayesian taxonomy classifier for 16S and ITS sequences. bioRxiv. 2016;PPR33123. 10.1101/074161.

[70] Lee T, Alemseged Y, Mitchell A. Dropping Hints: Estimating the diets of livestock in rangelands using DNA metabarcoding of faeces. Metabarcod Metagenom. 2018;2:e22467. 10.3897/mbmg.2.22467.

[71] Alberdi A, Garin I, Aizpurua O, Aihartza J. The foraging ecology of the Mountain Long-eared bat *Plecotus macrobullaris* revealed with DNA mini-barcodes. PLoS ONE. 2012;7:e35692. 10.1371/journal.pone.0035692. 22545129 10.1371/journal.pone.0035692 PMC3335802

[72] R Core Team. R: A Language and Environment for Statistical Computing. Version 4.3.2. Vienna: R Foundation for Statistical Computing; 2023.

[73] Corby-Harris V, Maes P, Anderson KE. The Bacterial Communities Associated with Honey Bee (*Apis mellifera*) Foragers. PLoS ONE. 2014;9(4):e95056. 10.1371/JOURNAL.PONE.0095056. 24740297 10.1371/journal.pone.0095056 PMC3989306

[74] Corby-Harris V, Snyder L, Meador C, Naldo R, Mott BM, Anderson KE. *Parasaccharibacter apium*, gen. nov., sp. nov., Improves Honey Bee (Hymenoptera: Apidae) Resistance to Nosema. J Econ Entomol. 2016;109:537–43. 10.1093/jee/tow012. 26875068 10.1093/jee/tow012

[75] Nevas M, Hielm S, Lindström M, Horn H, Koivulehto K, Korkeala H. High prevalence of Clostridium botulinum types A and B in honey samples detected by polymerase chain reaction. Int J Food Microbiol. 2002;72:45–52. 10.1016/s0168-1605(01)00615-8. 11843412 10.1016/s0168-1605(01)00615-8

[76] Piva S, Giacometti F, Marti E, Massella E, Cabbri R, Galuppi R, et al. Could honey bees signal the spread of antimicrobial resistance in the environment? Lett Appl Microbiol. 2020;70:349–55. 10.1111/lam.13288. 32096241 10.1111/lam.13288

[77] Audisio MC, Torres MJ, Sabaté DC, Ibarguren C, Apella MC. Properties of different lactic acid bacteria isolated from *Apis mellifera* L. bee-gut. Microbiol Res. 2011;166:1–13. 10.1016/j.micres.2010.01.003. 10.1016/j.micres.2010.01.003 20116222

[78] Kim PS, Shin NR, Kim JY, Yun JH, Hyun DW, Bae JW. *Acinetobacter apis* sp. nov., isolated from the intestinal tract of a honey bee, *Apis mellifera*. J Microbiol. 2014;52:639–645. 10.1007/s12275-014-4078-0. 10.1007/s12275-014-4078-0 25098562

[79] Álvarez Pérez S, Lievens B, Jacquemyn H, Herrera CM. *Acinetobacter nectaris* sp. nov. and *Acinetobacter boissieri* sp. nov., isolated from floral nectar of wild Mediterranean insect-pollinated plants. Int J Sys Evol Microbiol. 2013;63:1532–1539. 10.1099/ijs.0.043489-0. 10.1099/ijs.0.043489-0 22904213

[80] Fünfhaus A, Ebeling J, Genersch E. Bacterial pathogens of bees. Curr Op Insect Sci. 2018;26:89–96. 10.1016/j.cois.2018.02.008. 10.1016/j.cois.2018.02.008 29764667

[81] Edgar GJ. Population regulation, population dynamics and competition amongst mobile epifauna associated with seagrass. J Exp Marine Biol Ecol. 1990;144(2–3):205–34. 10.1016/0022-0981(90)90029-C.

[82] Hubbell SP. Neutral theory in community ecology and the hypothesis of functional equivalence. Funct Ecol. 2005;19(1):166–72. 10.1111/j.0269-8463.2005.00965.x.

[83] Salonen A, Ollikka T, Grönlund E, Ruottinen L, Julkunen-Tiitto R. Pollen analyses of honey from Finland. Grana. 2009;48(4):281–9. 10.1080/00173130903363550.

[84] Gillman LN, Wright SD. The influence of productivity on the species richness of plants: a critical assessment. Ecology. 2006;87(5):1234–43. 10.1890/0012-9658(2006)87[1234:TIOPOT]2.0.CO;2. 16761602 10.1890/0012-9658(2006)87[1234:tiopot]2.0.co;2

[85] Goldblatt P, Manning J. Plant diversity of the Cape region of Southern Africa. Ann Missouri Bot Gard. 2002;89(2):281–302. 10.2307/3298566.

[86] Bannister J, Vidal O, Teneb E, Sandoval V. Latitudinal patterns and regionalization of plant diversity along a 4270-km gradient in continental Chile. Austral Ecol. 2012;37:500–9. 10.1111/j.1442-9993.2011.02312.x.

[87] von Frisch, K. The Dance Language and Orientation of Bees. Cambridge: Harvard University Press; 1967.

[88] Waller GD. Evaluating responses of honey bees to sugar solutions using an artificial-flower feeder. Ann Entomol Soc America. 1972;65:857–62.

[89] Hung KLJ, Kingston JM, Lee A, Holway DA, Kohn JR. Non-native honey bees disproportionately dominate the most abundant floral resources in a biodiversity hotspot. Proc R Soc B. 2019;286:20182901. 10.1098/rspb.2018.2901. 30963829 10.1098/rspb.2018.2901 PMC6408903

[90] Rollin O, Bretagnolle V, Decourtye A, Aptel J, Michel N, Vaissière BE, et al. Differences Of floral resource use between honey bees and wild bees in an intensive farming system. Agriculture Ecosys Env. 2013;179(October):78–86. 10.1016/j.agee.2013.07.007.

[91] Urbanowicz C, Muñiz PA, McArt SH. Honey bees and wild pollinators differ in their preference for and use of introduced floral resources. Ecol Evol. 2020;10:6741–51. 10.1002/ece3.6417. 32724547 10.1002/ece3.6417 PMC7381584

[92] Tiusanen M, Becker-Scarpitta A, Wirta H. Distinct communities and differing dispersal routes in bacteria and fungi of honey bees, hives and flowers. Microb Ecol. 2024;87;100. 10.1007/s00248-024-02413-z. 10.1007/s00248-024-02413-z PMC11289361 39080099

[93] Fuhrman JA, Steele JA, Hewson I, Schwalbach MS, Brown MV, Green JL, et al. A latitudinal diversity gradient in planktonic marine bacteria. Proc Natl Acad Sci. 2008;105:7774–8. 10.1073/pnas.0803070105. 18509059 10.1073/pnas.0803070105 PMC2409396

[94] Kinlock NL, Prowant L, Herstoff EM, Foley CM, Akin-Fajiye M, Bender N, et al. Explaining global variation in the latitudinal diversity gradient: Meta-analysis confirms known patterns and uncovers new ones. Global Ecol Biogeo. 2017. 10.1111/geb.12665.

[95] Kwong WK, Moran NA. Gut microbial communities of social bees. Nat Rev Microbiol. 2016;14:374–84. 10.1038/nrmicro.2016.43. 27140688 10.1038/nrmicro.2016.43 PMC5648345

[96] McFrederick QS, Wcislo WT, Taylor DR, Ishak HD, Dowd SE, Mueller UG. Environment or kin: whence do bees obtain acidophilic bacteria? Mol Ecol. 2012;21(7):1754–68. 10.1111/j.1365-294X.2012.05496.x. 22340254 10.1111/j.1365-294X.2012.05496.x

[97] Olofsson TC, Vásquez A. Detection and identification of a novel lactic acid bacterial flora within the honey stomach of the honeybee *Apis mellifera*. Curr Microbiol. 2008;57(4):356–63. 10.1007/s00284-008-9202-0. 18663527 10.1007/s00284-008-9202-0

[98] Walter J, O’Toole PW. Microbe Profile: The Lactobacillaceae. Microbiology. 2023;169:001414. 10.1099/mic.0.001414. 38088348 10.1099/mic.0.001414 PMC10765037

[99] Girardin H, Albagnac C, Dargaignaratz C, Nguyen-The C, Carlin F. Antimicrobial activity of foodborne *Paenibacillus* and *Bacillus* spp. against *Clostridium botulinum*. J Food Protect. 2002;65:806–813. 10.4315/0362-028x-65.5.806. 10.4315/0362-028x-65.5.806 12030292

[100] Nielsen P, Sørensen J. Multi-target and medium-independent fungal antagonism by hydrolytic enzymes in *paenibacillus polymyxa* and *bacillus pumilus* strains from barley rhizosphere. FEMS Microbiol Ecol. 1997;22:183–92. 10.1111/j.1574-6941.1997.tb00370.x.

[101] Hubbell SP. Neutral theory and the theory of island biogeography. In: Losos JB, Ricklefs RE, editors. The theory of island biogeography revisited. Princeton: Princeton University Press; 2009. pp. 264–92.

