# Supplementary material for: The Latitudinal Biotic Interaction Hypothesis revisited: contrasting latitudinal richness gradients in actively vs. passively accumulated interaction partners of honey bees
Source: BMC Ecol Evol. 2025 Mar 17;25:24. doi: 10.1186/s12862-025-02363-1 (PMC11912709; doi:10.1186/s12862-025-02363-1)
Supplement: Supplementary file 1 — Supplementary Material 1. [file 12862_2025_2363_MOESM1_ESM.pdf]

The Latitudinal Biotic Interaction Hypothesis revisited: Contrasting latitudinal diversity gradients in actively vs. passively accumulated interaction partners of honey bees Supplemental information document

Alyssa R. Cirtwill, Tomas Roslin, Pablo Peña-Aguilera, Agathe Agboto, William Bercê, Svetlana N. Bondarchuk, Robert Brodschneider, Behzad Heidari, Camara Kaizirege, Justine Muhoro Nyaga, Ojonugwa Ekpah, Gonzalo Ossa Gomez, Claudia Paz, Christian Pirk, Amir Salehi-Najafabadi, Anneli Salonen, Chantal Soloniaina, Helena Wirta*

*Corresponding author and lead contact: helena.wirta@umu.se

Table of contents

Text S1: Sample and laboratory details

- Table S1: Samples per country
- Table S2: Primers used in DNA amplification

Text S2: Testing independence of predictors

- Table S3: χ^2 values for correlation of predictors in the plant dataset.
- Table S4: χ^2 values for correlation of predictors in the bacteria dataset.

Text S3: Testing dependence of diversity on reads

- Table S5: F-statistics and p-values for quasi-Poisson regressions relating number of taxa to number of reads.

Text S4: Testing the effect of other sample characteristics

- Methods
- Results: Year of sampling
  - Table S6: Coefficients and F-statistics for regressions relating diversity of plants and bacteria in honey samples to absolute latitude, year of sampling, and their interaction.
  - Figure S1: Variation in the relationship between diversity of plants and bacteria in honey samples and absolute latitude across years.
- Results: Number of hives
  - Table S7: Coefficients and F-statistics for regressions relating diversity of plants and bacteria in honey samples to absolute latitude, number of hives included in a sample, and their interaction.
  - Figure S2: Variation in the relationship between diversity of plants and bacteria in honey samples and absolute latitude across numbers of hives included in a sample.
- Results: Hive type
  - Table S8: Coefficients and F-statistics for regressions relating diversity of plants and bacteria in honey samples to absolute latitude, hive type, and their interaction.
  - Figure S3: Variation in the relationship between diversity of plants and bacteria in honey samples and absolute latitude across hive types.

- Results: Method of honey extraction

  – Table S9: Coefficients and F-statistics for regressions relating diversity of plants and bacteria in honey samples to absolute latitude, method of honey extraction, and their interaction.

  – Figure S4: Variation in the relationship between diversity of plants and bacteria in honey samples and absolute latitude across methods of honey extraction.

Text S5: Relating genera and families to latitude

– Table S10: Coefficients, F-statistics, and p-values for the absolute latitude term in quasi-Poisson regressions relating number of taxa to absolute latitude and log(reads).

– Table S11: Coefficients, F-statistics, and p-values for the log(reads) term in quasi-Poisson regressions relating number of taxa to absolute latitude and log(reads).

– Figure S5: Relationship between absolute latitude and diversity of plants and bacteria in honey samples for ZOTU, genera, and families.

Text S6: Exploring effects of geographical aggregation

- Methods

- Results

– Table S12: Summary statistics and number of subset models giving matching conclusions to the full-dataset models

Text S7: Latitudinal trends in niche breadth in the honey bee's native range

– Table S13: Summary statistics and number of subset models giving matching conclusions to the full-dataset models

S1. Sample and laboratory details

Table S1: Number of samples per country with DNA successfully amplifed. These may differ between taxonomic groups if only plant or only bacterial DNA was recovered from a given sample.

Country	N(Plant)	N(Bacteria)	Country	N(Plant)	N(Bacteria)
Austria	17	17	Kenya	8	9
Benin	28	30	Latvia	7	7
Brazil	4	4	Madagascar	25	25
Bulgaria	10	10	Malta	2	2
Canada	5	5	Netherlands	1	1
Chile	16	16	Nigeria	17	17
DRC	1	0	Russia	8	8
Finland	28	28	South-Africa	20	20
France	1	1	Spain	17	17
Germany	1	1	Tanzania	12	12
Guatemala	1	1	Ukraine	2	2
Iran	20	20			

Table S2: Primers used in DNA extraction and amplification for bacteria and vascular plants.

gene region	taxa	primer name	primer sequence	sequence order	references
16S	bacteria	tagF_16S_515FB	tcgtcggcagcgtcagatgtg-tataagagacagGT-GYCAGCMGCCGCG-GTAA	5'-3'	Caporaso et al. 2011, Walters et al. 2015
16S	bacteria	tagR_16S_806RB	gtctcgtgggctcggagatgtg-tataagagacagGGACTAC-NVGGGTWTCTAAT	5'-3'	Caporaso et al. 2011, Walters et al. 2015
ITS2	vascular plants	tagF_ITS2-F	tcgtcggcagcgtcagatgtg-tataagagacagATGCGAT-ACTTGGTGTGAAT	5'-3'	Chen et al. 2010, White et al. 1990
ITS2	vascular plants	tagR_ITS2-R	gtctcgtgggctcggagatgtg-tataagagacagTCCTC-CGCTTATTGATATGC	5'-3'	Chen et al. 2010, White et al. 1990

S2. Testing independence of predictors

We collected information on month and year of sampling, number of hives, number of beekeepers, type of hive, method of honey extraction, country, and approximate latitude and longitude. It is highly likely that not all of these predictors are independent and can be removed from our analyses. We therefore tested whether each pair of predictors was independent. We tested whether latitude and longitude varied with other predictors using an analysis of variance of a model relating latitude or longitude (continuous variables) to the other predictor (categorical, except when relating latitude to longitude). A significant result of these tests indicates non-independence; all tests were fit using the R [1] base functions 'lm' and 'aov'. For all other pairs of (categorical) predictors, we tested for non-independence using a series of Pearson's χ^2 tests (fit using the R [1] base function 'chisq.test'). For this test, a significant result indicates independence.

Where we identified non-independent pairs of predictors, we considered only one of the pair in all future analyses. We prioritised first continuous predictors (latitude and longitude) and then categorical predictors with a more even distribution of samples between categories (e.g., number of beekeepers was less-preferred because 242 samples were collected by one beekeeper and the remaining nine by more than two). Month of sampling was not considered as a predictor because of the difficulty in interpreting month across a global dataset: both seasonality and time since previous harvest will vary both with month and country. As all other predictors were not independent of latitude, we therefore consider latitude alone in further analyses.

Table S3: Testing for independence of predictors in plant dataset. Note that latitude and longitude are continuous predictors while all others are treated as categorical. Entries in bold are non-independent (p<0.05 for anova and p>0.05 for χ^2 tests). Values for models relating latitude and longitude to other predictors are F-statistics, otherwise Pearson's contingency coefficient. For two cases these values could not be calculated (marked with *) and instead χ^2 values are given.

	Lat	Lon	Year	Country	Type	Method	N. Hives
Longitude	**16.4**						
Year	**4.59**	**4.40**					
Country	**2340**	**3543**	**0.984**				
Type	**32.6**	**10.1**	**0.560**	**0.983**			
Method	**47.2**	**7.40**	**0.557**	**0.958**	**0.948**		
Hives	**7.06**	**18.5**	**0.687**	535*	**0.753**	**0.739**	
Beekeepers	**5.22**	**4.23**	**0.300**	**0.695**	0.136	0.176	0.304

Table S4: Testing for independence of predictors in bacterial dataset. Note that latitude and longitude are continuous predictors while all others are treated as categorical. Entries in bold are non-independent (p<0.05 for anova and p>0.05 for χ^2 tests). Values for models relating latitude and longitude to other predictors are F-statistics, otherwise Pearson's contingency coefficient. For two cases these values could not be calculated (marked with *) and instead χ^2 values are given.

	Lat	Lon	Year	Country	Type	Method	N. Hives
Longitude	**16.6**						
Year	**4.88**	**4.40**					
Country	**2370**	**3770**	**0.984**				
Type	**32.8**	**10.4**	**0.548**	**0.982**			
Method	**47.5**	**7.58**	**0.547**	**0.955**	**0.948**		
Hives	**7.04**	**18.5**	**0.688**	535*	**0.753**	**0.739**	
Beekeepers	**5.24**	**4.23**	**0.292**	**0.696**	0.138	0.178	0.304

S3. Testing dependence of diversity on reads

For plants and bacteria, we first explored whether diversity (number of families, genera, and zotu) was related to the total number of reads obtained in a sample. Ideally, this relationship would reach an asymptote indicating a number of reads which is sufficient to adequately capture the true diversity. We therefore tested whether diversity increased with increasing number of reads in each dataset using a quasi-Poisson regression, using ANOVA to evaluate significance. All regressions were conducted using the R [1] base function 'glm'. We do not observe such an asymptote in our data for bacteria or plant ZOTUs, though diversity is not related to the number of reads for plant genera or families (Table S5). As, in general, we expect that samples from which we obtained more reads to more-fully capture the true diversity of a sample (whether that diversity is high or low), we included number of reads as weights in all models relating diversity to latitude or other predictors.

Table S5: Mean numbers of reads and taxa in each of our datasets, together with F-statistics and p-values for quasi-Poisson regressions relating number of taxa to number of reads. Numbers of reads varies slightly between dataset depending on the number of reads which could be identified.

	Plants				Bacteria			
Level	Reads	Taxa	F	p	Reads	Taxa	F	p
ZOTU	8822	33.6	16.5	**<0.001**	9680	54.9	4.06	**0.045**
Genera	8822	11.2	1.35	0.246	9680	15.7	16.2	**<0.001**
Families	8822	8.60	1.31	0.253	9680	14.9	15.1	**<0.001**

S4. Testing the effect of other sample characteristics

Although all other predictors we collected were significantly correlated with latitude (Text *S2*), we present tests for whether the diversity-latitude relationship varies between levels of other predictors for illustration purposes. Note that, in general, significant interactions between latitude and other predictors are due to strong clustering of some values of the other predictor at particular latitude.

Methods

After identifying the major trends over latitude, we tested whether these trends varied with year of sampling, number of hives, hive type, and method of harvest. As all of these predictors are significantly correlated with latitude, we did not combine multiple predictors into a single model. We therefore fit general linear models including absolute latitude, the other predictor (i.e., number of hives or hive type or method of harvest), and the interaction between latitude and the other predictor. To make interpretation of the categorical variables clearer, we did not include a separate intercept term in these models. This amounts to fitting a separate intercept and slope for each level of the factorial predictor. Note that levels of the other predictors are not distributed evenly over latitude (Text *S2*). Significance of each term in these models was evaluated using ANOVA (R [1] base function 'aov').

Results

Year of sampling

Table S6: Coefficients, degrees of freedom, F-statistics, and p-values for quasi-Poisson regressions of diversity against absolute latitude, year of sampling (numeric), and their interaction. log(reads) was included as a term in the regression.

	ZOTU			Genera			Families		
**Plants**	df	F	P	df	F	P	df	F	P
log(reads)	1, 247	23.6	**<0.001**	1, 247	7.36	**0.007**	1, 247	8.73	**0.003**
abs(lat)	1, 247	856	**<0.001**	1, 247	897	**<0.001**	1, 247	1020	**<0.001**
Year	1, 247	92.4	**<0.001**	1, 247	148	**<0.001**	1, 247	256	**<0.001**
abs(lat):Year	1, 247	11.2	**<0.001**	1, 247	1.81	0.180	1, 247	3.41	0.066
**Bacteria**	df	F	P	df	F	P	df	F	P
log(reads)	1, 249	6.96	**0.009**	1, 249	0.127	0.722	1, 249	0.044	0.834
abs(lat)	1, 249	283	**<0.001**	1, 249	292	**<0.001**	1, 249	321	**<0.001**
Year	1, 249	367	**<0.001**	1, 249	368	**<0.001**	1, 249	313	**<0.001**
abs(lat):Year	1, 249	0.012	0.913	1, 249	3.13	0.078	1, 249	4.40	**0.037**

Number of hives

Trends of diversity over latitude varied ideosyncratically with number of hives (Fig. S2). In particular, bacterial diversity of samples pooled from 3-5 hives increased with latitude,

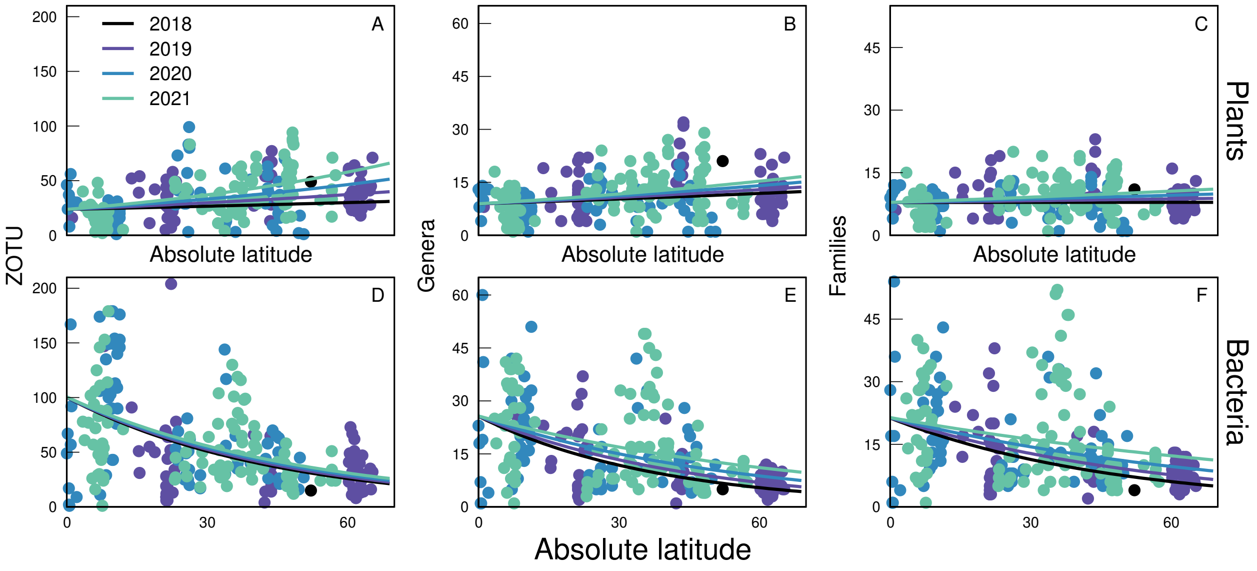

Figure S1: The slope of diversity vs. latitude was similar between honey samples collected in different years, but significantly steeper in later years for plant ZOTU and bacteria families. For other taxonomic group and level combinations, there was no significant difference in slopes between years of sampling. Slopes shown are for the mean number of reads per sample for each taxonomic group.

contrary to other samples. Most such samples (20/31) were from Iran, twith the remaining samples coming from Finland (4), Austria (2), Benin (2), Canada (2), and South Africa (1). As such, fitting of latitudinal trends for this group is likely to be poor and the exceptional slope essentially reflects higher observed diversity in Iran.

Hive type

Modern hives were, by far, the most common hive type. Top-bar hives were rare and tended to be found in the tropics, up to about 25° North of South. Many tropical hives

Table S7: Coefficients, degrees of freedom, *F*-statistics, and *p*-values for quasi-Poisson regressions of diversity against absolute latitude, number of hives (categorical), and their interaction. log(reads) was included as a term in the regression.

	ZOTU			Genera			Families		
**Plants**	df	F	P	df	F	P	df	F	P
log(reads)	1, 186	11.5	**<0.001**	1, 186	2.17	0.142	1, 186	3.17	0.077
abs(lat)	1, 186	735	**<0.001**	1, 186	777	**<0.001**	1, 186	909	**<0.001**
N. Hives	7, 186	14.5	**<0.001**	7, 186	17.1	**<0.001**	7, 186	30.5	**<0.001**
abs(lat):N. Hives	6, 186	0.678	0.668	6, 186	0.245	0.961	6, 186	0.337	0.917
**Bacteria**	df	F	P	df	F	P	df	F	P
log(reads)	1, 186	1.61	0.206	1, 186	0.122	0.727	1, 186	0.223	0.637
abs(lat)	1, 186	238	**<0.001**	1, 186	228	**<0.001**	1, 186	257	**<0.001**
N. Hives	7, 186	43.4	**<0.001**	7, 186	42.9	**<0.001**	7, 186	38.2	**<0.001**
abs(lat):N. Hives	6, 186	1.99	0.069	6, 186	0.753	0.607	6, 186	0.810	0.564

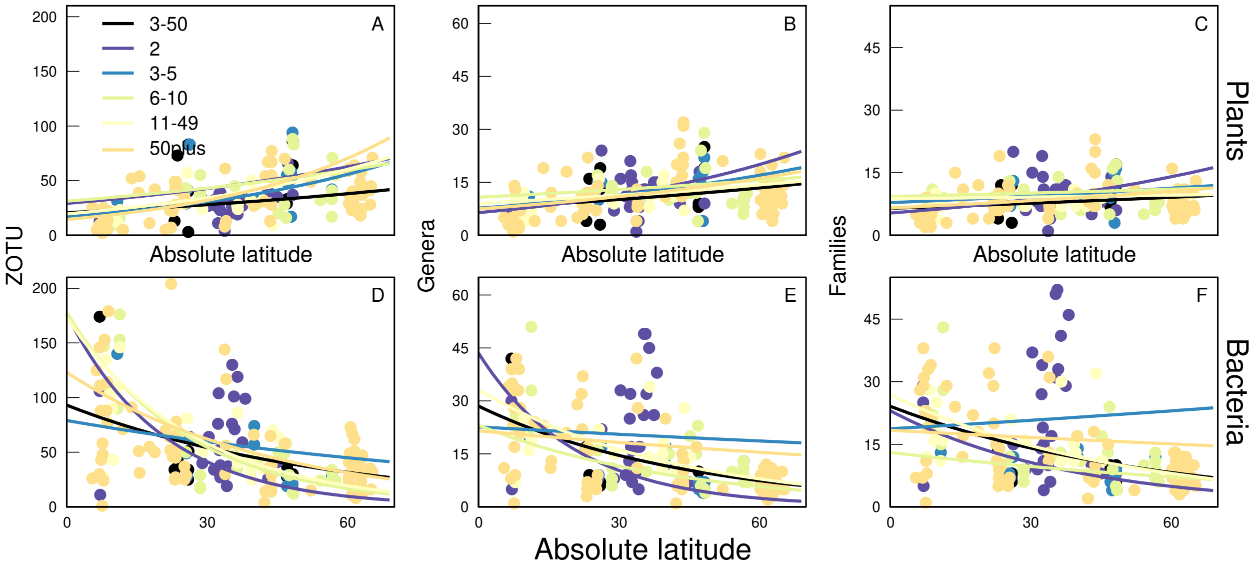

Figure S2: The slope of diversity vs. latitude varied with number of hives. Slopes shown are for the mean number of reads per sample for each taxonomic group.

also had an unknown hive type, which could represent modern or top-bar hives or traditional log hives. Thus, hive type is strongly linked to latitude and unlikely to provide much independent information in our dataset. Indeed, there was no significant interaction between latitude and hive type for any taxonomic group or level.

Table S8: Coefficients, degrees of freedom, F-statistics, and p-values for quasi-Poisson regressions of diversity against absolute latitude, hive type (categorical; modern, top-bar, or unknown), and their interaction. log(reads) was included as a term in the regression.

	ZOTU			Genera			Families		
**Plants**	df	F	P	df	F	P	df	F	P
log(reads)	1, 244	19.8	**<0.001**	1, 244	5.04	**0.026**	1, 244	5.79	**0.017**
abs(lat)	1, 244	850	**<0.001**	1, 244	915	**<0.001**	1, 244	1050	**<0.001**
Hive type	3, 244	34.6	**<0.001**	3, 244	54.3	**<0.001**	3, 244	93.2	**<0.001**
abs(lat):Hive type	2, 244	1.92	0.149	2, 244	0.080	0.924	2, 244	0.594	0.553
**Bacteria**	df	F	P	df	F	P	df	F	P
log(reads)	1, 246	8.93	**0.003**	1, 246	0.223	0.637	1, 246	0.037	0.848
abs(lat)	1, 246	309	**<0.001**	1, 246	292	**<0.001**	1, 246	315	**<0.001**
Hive type	3, 246	142	**<0.001**	3, 246	125	**<0.001**	3, 246	103	**<0.001**
abs(lat):Hive type	2, 246	0.272	0.762	2, 246	0.180	0.835	2, 246	0.030	0.971

Method of honey extraction

As with hive type, method of honey extraction was strongly dependent on latitude. Most samples from temperate latitudes (>30°) were extracted by centrifuging, while squeezing and pressing were more common in the tropics. Samples extracted using an unknown method were also more likely to come from very low latitudes, making the estimation of effects of extraction on observed diversity unreliable for tropical samples.

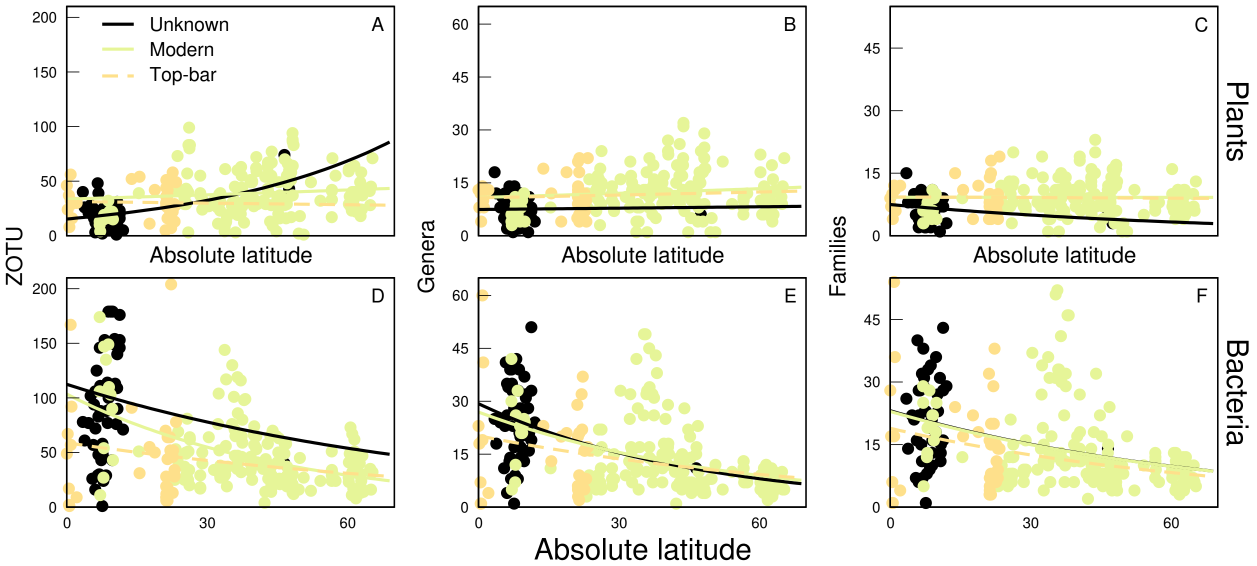

Figure S3: The slope of diversity vs. latitude varied with hive type. Slopes are shown for the mean number of reads per sample in each taxonomic group.

Table S9: Coefficients, degrees of freedom, F-statistics, and p-values for quasi-Poisson regressions of diversity against absolute latitude, method of honey extraction (categorical), and their interaction. log(reads) was included as a term in the regression.

	ZOTU			Genera			Families		
**Plants**	df	F	P	df	F	P	df	F	P
log(reads)	1, 242	16.9	**<0.001**	1, 242	4.60	**0.033**	1, 242	4.97	**0.027**
abs(lat)	1, 242	872	**<0.001**	1, 242	920	**<0.001**	1, 242	1050	**<0.001**
Method	4, 242	28.8	**<0.001**	4, 242	41.8	**<0.001**	4, 242	71.1	**<0.001**
abs(lat):Method	3, 242	2.30	0.078	3, 242	0.164	0.921	3, 242	0.369	0.776
**Bacteria**	df	F	P	df	F	P	df	F	P
log(reads)	1, 244	13.8	**<0.001**	1, 244	0.449	0.503	1, 244	0.002	0.961
abs(lat)	1, 244	339	**<0.001**	1, 244	298	**<0.001**	1, 244	323	**<0.001**
Method	4, 244	121	**<0.001**	4, 244	97.5	**<0.001**	4, 244	81.5	**<0.001**
abs(lat):Method	3, 244	1.25	0.292	3, 244	0.227	0.878	3, 244	0.107	0.956

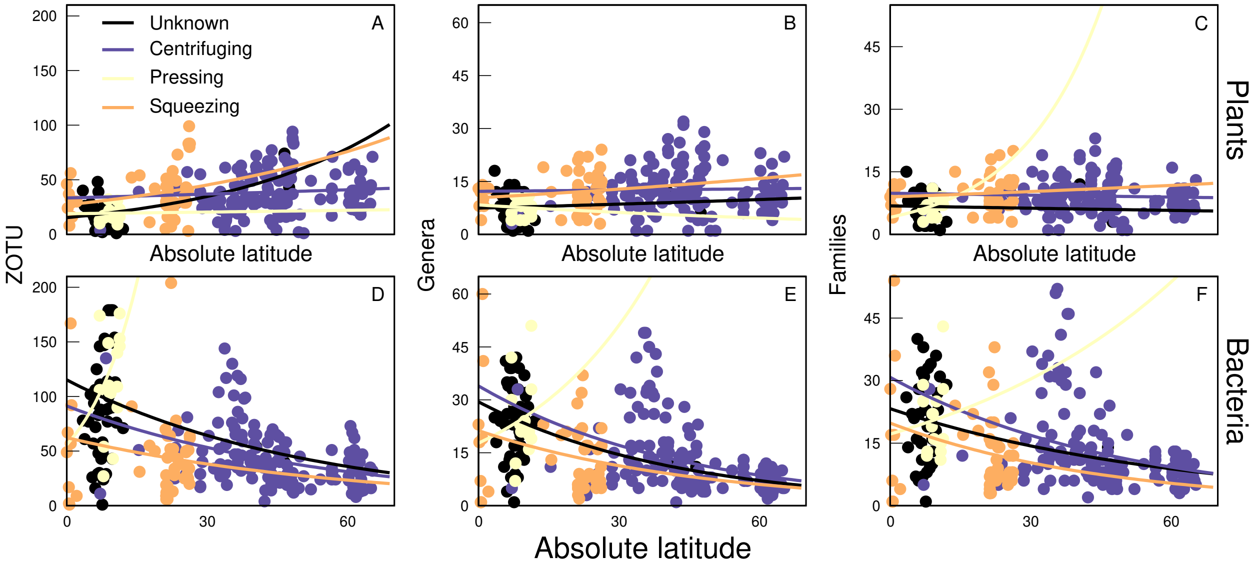

Figure S4: The slope of diversity vs. latitude varied with method of honey extraction. Slopes are shown for the average number of reads per sample for each taxonomic group.

S5. Relating genera and families to latitude

For ZOTU-level results, see *Main Text*.

Table S10: Coefficients, F-statistics, and p-values for the absolute latitude term in quasi-Poisson regressions of diversity against absolute latitude and the log of total number of reads (see Table S11 for coefficients of total reads). Note that in a quasi-Poisson regression, $y = e^{intercept+\beta}$.

	ZOTU			Genera			Families		
Group	β	F	p	β	F	p	β	F	p
Plants	**9.78×10^{-3}**	**42.8**	**<0.001**	**7.09×10^{-3}**	**23.4**	**<0.001**	**2.83×10^{-3}**	**6.26**	**0.013**
Bacteria	**-2.07×10^{-2}**	**61.3**	**<0.001**	**-1.79×10^{-2}**	**58.8**	**<0.001**	**-1.33×10^{-2}**	**35.4**	**<0.001**

Table S11: Coefficients, F-statistics, and p-values for the log(reads) term in the regressions described in Table S10.

	ZOTU			Genera			Families		
Group	β	F	p	β	F	p	β	F	p
Plants	**3.03×10^{-1}**	**22.7**	**<0.001**	**1.33×10^{-1}**	**7.33**	**0.007**	**1.26×10^{-1}**	**8.65**	**0.004**
Bacteria	**2.31×10^{-1}**	**7.00**	**0.009**	**4.35×10^{-2}**	0.137	0.712	**-4.87×10^{-3}**	0.037	0.847

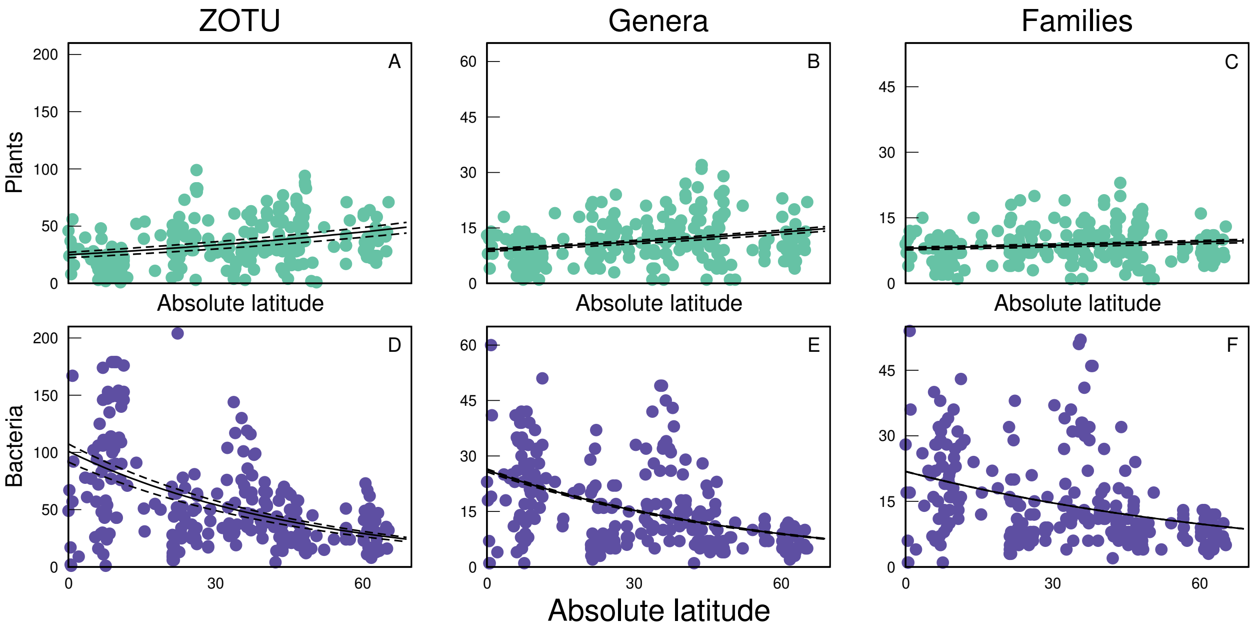

Figure S5: Bacterial diversity decreased towards the poles while plant diversity generally increased towards the poles. There was no significant trends of plant family diversity over latitude. Black lines indicate fits of quasi-Poisson regressions of diversity against absolute latitude and the log of total reads per sample. The solid line represents the fit for the mean number of reads for each taxonomic group and the dotted lines represent the 25% and 75% quantiles.

S6. Exploring effects of geographical aggregation

Methods

Our data are unevenly distributed across countries, as seen in Table *S1* and Fig. 1 (Main Text). To test whether this geographic aggregation of samples has influenced our results, we conducted a rarefaction analysis in which we re-fit our models relating niche breadth to absolute latitude and log(reads) using subsets of the data. As with the full-dataset models, we fit separate quasi-Poisson regression models for plant or bacteria ZOTUs, genera, or families using the R [1] function 'glm'.

To construct data subsets, we first divided our data between countries with the median or fewer number of samples (8 for plants, 9.5 –rounded down to 9– for bacteria) and those with more than the median number of samples. For countries with relatively few samples as defined above, we included all samples in every data subset. These countries were: Brazil, Canada, Democratic Republic of Congo, France, Germany, Guatemala, Kenya, Latvia, Malta, Netherlands, Russia, and Ukraine. For countries with relatively many samples, we randomly selected 8 (for plants) or 9 (for bacteria) samples per country to include in the data subset. This reduces the influence these countries (Austria, Benin, Bulgaria, Chile, Finland, Iran, Madagascar, Nigeria, South Africa, Spain, Tanzania) have on the models. Note that both countries with many samples and countries with few samples are widely-distributed over latitude and between continents.

We repeated this rarefaction procedure 1000 times for each combination of taxonomic group (plants or bacteria) and level (ZOTU, genera, or families) and fit a quasi-Poisson regression relating niche breadth to absolute latitude and log(reads) to each data subset. In each case, we recorded the estimated coefficients for each term and tested the significance of the absolute latitude and log(reads) terms using ANOVA, as in the main models. We then counted the number of cases in which the significance obtained using the data subset matched that obtained using the full dataset. This gives us a measure of the extent to which the dominance of particular countries in our full dataset may have influenced our conclusions.

Results

Overall, the results of models fit using more geographically-even subsets of our data were qualitatively identical to those obtained using the full dataset (Table S12. From this, we conclude that the uneven number of samples per country did not significantly affect our conclusions. Only two terms, in different models, did not have overwhelmingly the same significance as when using the full dataset. The log(reads) term in the model for niche breadth defined using bacterial ZOTUs was significant when using the full dataset and in over half of subsets; as the log(reads) term was only included in order to account for potential effects of the number of reads on the observed bacterial diversity, we do not consider this variability to be important. More importantly, the absolute latitude term in the model defining niche breadth using number of plant families was significant when using the full dataset but only in a minority of subsets. We therefore conclude that this 'significant' effect is likely to be due to the geographical aggregation of our samples rather

than a true trend. This is not entirely unexpected as the slope of this relationship, even when significant, was very close to zero.

Table S12: Ranges of estimated coefficients (Estimate), F-statistics and p-values for ANOVA tests of significance, and the number of subset models for which significance matches that from the full dataset (Matches). We show these values for plants (P) and bacteria (B) and ZOTUs, Genera, and Families. Note that the only term for which signficance does not generally match that obtained from the full dataset is the relationship between plant family richness in honey and absolute latitude (indicated in bold).

Tax.	Level	Term	Estimate		F		p		Matches
P	ZOTU	abs(Lat)	6.47E-03	1.29E-02	1.06E+01	3.54E+01	0.000	0.001	1000/1000
P	ZOTU	log(Reads)	2.51E-01	4.84E-01	1.08E+01	3.87E+01	0.000	0.001	1000/1000
P	Genera	abs(Lat)	2.73E-03	1.01E-02	2.58E+00	2.18E+01	0.000	0.110	997/1000
P	Genera	log(Reads)	1.35E-01	3.21E-01	3.63E+00	2.22E+01	0.000	0.059	998/1000
**P**	**Famlies**	**abs(Lat)**	-1.05E-03	5.09E-03	1.44E-03	7.85E+00	0.006	0.970	**72/1000**
P	Famlies	log(Reads)	1.08E-01	2.78E-01	2.63E+00	2.36E+01	0.000	0.107	995/1000
B	ZOTU	abs(Lat)	-2.26E-02	-1.27E-02	1.30E+01	4.89E+01	0.000	0.000	1000/1000
B	ZOTU	log(Reads)	6.57E-02	3.73E-01	2.41E-01	1.09E+01	0.241	10.918	678/1000
B	Genera	abs(Lat)	-2.12E-02	-1.26E-02	1.33E+01	5.15E+01	0.000	0.000	1000/1000
B	Genera	log(Reads)	-9.44E-02	1.93E-01	1.03E-09	2.63E+00	0.107	1.000	1000/1000*
B	Famlies	abs(Lat)	-1.71E-02	-8.65E-03	7.76E+00	3.73E+01	0.000	0.006	1000/1000
B	Famlies	log(Reads)	-1.40E-01	1.34E-01	9.21E-08	2.54E+00	0.113	1.000	1000/1000*

Note that these terms were non-significant when using the full data

S7: Considering only samples within the native range

To control for the possibility that latitudinal niche breadth patterns differ between the historical native range of *Apis mellifera* and areas where honeybees have been more recently introduced, we divided our samples into 'native' and 'exotic' groups based countries included in the native range defined in Requier *et al.* [2], Tihelka *et al.* [3]. We then re-fit our models using only samples taken from the native range of *A. mellifera*. Fitting these models using samples from the exotic range is not appropriate because of the small number of samples in this group (62) and the overwhelming dominance of samples from Finland (28) and Russia (8), leaving only 26 samples from the Americas to represent most of the exotic range. Our results based only on samples located within the native range of honeybees are extremely similar to those obtained using our full dataset. Given that the majority of our samples (189 for plants, 191 for bacteria) were included in this 'native' group, this similarity is not surprising.

Table S13: Estimated coefficients (Estimate) and F-statistics and p-values for ANOVA tests of significance for models relating niche breadth defined using plant or bactera ZOTU, genera, or families, using only samples collected within the native range of *A. mellifera*. In all cases, our results are qualitatively identical to those obtained using our full dataset.

Taxon	Level	Term	Estimate	F	p
Plants	ZOTU	Abslat	1.92E-02	72.9	<0.001
Plants	ZOTU	log(Reads	3.47E-01	17.5	<0.001
Plants	Genera	Abslat	1.40E-02	46.3	<0.001
Plants	Genera	log(Reads	1.42E-01	5.58	0.019
Plants	Families	Abslat	8.48E-03	22.0	<0.001
Plants	Families	log(Reads	1.26E-01	6.32	0.013
Bacteria	ZOTU	Abslat	-2.30E-02	39.2	<0.001
Bacteria	ZOTU	log(Reads	2.73E-01	8.13	0.005
Bacteria	Genera	Abslat	-1.70E-02	27.9	<0.001
Bacteria	Genera	log(Reads	9.82E-02	1.05	0.307
Bacteria	Families	Abslat	-1.20E-02	14.5	<0.001
Bacteria	Families	log(Reads	4.63E-02	0.210	0.648

References

[1] R Core Team (2023). *R: A Language and Environment for Statistical Computing. Version 4.3.2.* R Foundation for Statistical Computing, Vienna, Austria.

[2] Requier, F., Garnery, L., Kohl, P., Njovu, H., Pirk, C., Crewe, R. & Steffan-Dewenter, I. (2019). The conservation of native honey bees is crucial. *Trends Ecol Evol*, 34, 789–798.

[3] Tihelka, E., Cai, C., Pisani, D. & Donoghue, P.C.J. (2020). Mitochondrial genomes illuminate the evolutionary history of the western honey bee (*Apis mellifera*). *Sci Rep*, 10.
